# Calorimetric and Dielectric Investigations of Epoxy-Based Nanocomposites with Halloysite Nanotubes as Nanofillers

**DOI:** 10.3390/polym13101634

**Published:** 2021-05-18

**Authors:** Hassan Omar, Glen J. Smales, Sven Henning, Zhi Li, De-Yi Wang, Andreas Schönhals, Paulina Szymoniak

**Affiliations:** 1 Bundesanstalt für Materialforschung und -prüfung (BAM), Unter den Eichen 87, 12205 Berlin, Germany; hassan.omar@bam.de (H.O.); glen-jacob.smales@bam.de (G.J.S.); andreas.schoenhals@bam.de (A.S.); 2 Fraunhofer-Institut für Mikrostruktur von Werkstoffen und Systemen (IMWS), Walter-Hülse-Str. 1, 06120 Halle, Germany; sven.henning@imws.fraunhofer.de; 3 IMDEA Materials Institute, C/Eric Kandel 2, 28906 Getafe, Spain; zhi.li@cqjtu.edu.cn (Z.L.); deyi.wang@imdea.org (D.-Y.W.)

**Keywords:** epoxy nanocomposites, halloysite nanotubes, X-ray scattering, differential scanning calorimetry, broadband dielectric spectroscopy, flash DSC, rigid amorphous fraction

## Abstract

Epoxy nanocomposites are promising materials for industrial applications (i.e., aerospace, marine and automotive industry) due to their extraordinary mechanical and thermal properties. Here, the effect of hollow halloysite nanotubes (HNT) on an epoxy matrix (Ep) was the focus of the study. The structure and molecular mobility of the nanocomposites were investigated using a combination of X-ray scattering, calorimetry (differential (DSC) and fast scanning calorimetry (FSC)) and dielectric spectroscopy. Additionally, the effect of surface modification of HNT (polydopamine (PDA) and Fe(OH)_3_ nanodots) was considered. For Ep/HNT, the glass transition temperature (*T_g_*) was decreased due to a nanoparticle-related decrease of the crosslinking density. For the modified system, Ep/m-HNT, the surface modification resulted in enhanced filler–matrix interactions leading to higher *T_g_* values than the pure epoxy in some cases. For Ep/m-HNT, the amount of interface formed between the nanoparticles and the matrix ranged from 5% to 15%. Through BDS measurements, localized fluctuations were detected as a β- and γ-relaxation, related to rotational fluctuations of phenyl rings and local reorientations of unreacted components. A combination of calorimetry and dielectric spectroscopy revealed a dynamic and structural heterogeneity of the matrix, as confirmed by two glassy dynamics in both systems, related to regions with different crosslinking densities.

## 1. Introduction

Polymer nanocomposites (PNCs) are systems composed of two or more components where the matrix is a polymer and the incorporated nanofillers have at least one dimension on the nanometer scale (<100 nm) [1]. PNCs have attracted considerable attention due to their improved properties (e.g., mechanical strength, toughness, electric and thermal conductivity and flame retardancy) compared to the unfilled polymer [2]. These improved properties can be achieved with low filler content, so that the system retains its low weight and ease of processability [2,3]. Additionally, by varying the type of filler and polymer matrix, and thereby the polymer–filler interactions, the properties of the PNC system can be tuned for specific purposes, thus providing the possibility of tunable nanocomposite systems [4].

The performance of a PNC, in parallel with the characteristics of the used components, depends on the size, the aspect ratio, the surface area and the weight fraction of the nanofiller. However, the most important factors are the dispersion of the nanofiller (filler–filler interactions) and the interfacial region formed between the bulk matrix and the nanofiller (filler–polymer interactions) [5]. The dispersion of nanofillers can be homogenous in the matrix but might also be associated with the formation of agglomerates. This results from short-range interactions such as van der Waals forces between the primary particles [6]. Aggregation can be controlled to a certain extent by employing specific sample preparation steps. The use of severe shear stress and surface modification of the filler have been shown to result in a homogenous particle dispersion in the polymer matrix [7]. On the one hand, a good dispersion of nanofillers has resulted in improved diffusion of solvents, heat deflection temperature and other thermal properties [8]. Moreover, aggregation can introduce defects and stress concentrations that have adverse effects on the mechanical properties [9]. On the other hand, aggregation has been shown to be related to a reinforcement of PNC properties in some cases [10].

Furthermore, “interfacial properties” have been found to have a significant contribution to the properties of PNCs. Due to the reduced dimensions of the filler, resulting in a high surface to volume ratio, there is an increased volume of interfacial area. For non-repulsive filler–matrix interactions, polymer segments are physically adsorbed to the nanofiller, with the formed interphase commonly referred to as the rigid amorphous fraction (RAF) [11]. The RAF can also be formed through chemical bonding of the nanoparticle surface to the matrix. The properties of the RAF may differ from those of the bulk matrix such as conformations, density and molecular mobility. The term “rigid” refers to a restricted or even immobilized segmental mobility in the interface compared to the bulk matrix [12]. The different molecular mobility at the interface, compared to the bulk matrix, can be investigated using broadband dielectric spectroscopy (BDS) in some cases [11,12,13,14]. Moreover, BDS allows for the investigation of the dynamical processes of PNCs in dependence on the concentration of the nanofiller on spatial length scales ranging from local to segmental fluctuations in a broad frequency range (mHz to GHz) [15]. Furthermore, one of the most useful techniques used to investigate filler-related effects on the polymer matrix is calorimetry. The glass transition temperature (*T_g_*) can be investigated using differential scanning calorimetry [16] and fast scanning calorimetry [17]. Moreover, by analyzing the change in the specific heat capacity at the glass transition (calorimetric strength) using modulated DSC (TMDSC) [18], the amount of RAF and/or the number of mobilized segments due to the nanofiller in the system can be quantified [19].

In this study, two epoxy nanocomposite systems based on bisphenol-A-diglycidyl ether (DGEBA) were investigated. Epoxies were formed during a curing reaction, resulting in a crosslinked structure, which could be altered depending on the reactants and curing time. These systems have various applications in adhesives, coatings, electronics and as construction materials in automotive as well as aerospace industries [5,20,21]. They have good mechanical strength, Young’s modulus and can be tailored for specific applications by tuning the crosslinking density. However, due to their brittleness and relatively high flammability, epoxy-based materials are commonly reinforced with inorganic particles to improve such properties [22,23].

Epoxy-based materials have been reinforced with nanoparticles (NPs), such as silica, barium titanate [24,25] or boehmite NPs [26,27], layered materials [11,28], polyhedral oligomeric silsesquioxane (POSS) [29,30] and carbon nanotubes (CNT) [31,32], to achieve improved properties compared to the unfilled epoxy. CNTs have been used as a nanofiller due to their high aspect ratio, having a significantly larger length compared to the inner and outer diameters, ease of processing and their ability to be surface modified [33]. In addition, the incorporation of CNTs leads to enhancements in several properties of PNCs, such as increased mechanical, electrical and thermal characteristics. Recently, halloysite nanotubes (HNT) have gained attention as a cheaper, less toxic and more environmentally friendly alternative to CNTs, while possessing a similar crystal structure and aspect ratio [7,34]. The literature shows that the incorporation of HNTs into a polymer matrix can result in improvements in thermal and mechanical stability, biocompatibility, flame retardancy and waterproof behavior, among other properties [35,36,37,38,39]. Li et al. showed an increase in both the storage modulus and tensile strength for an epoxy nanocomposite containing HNT, compared to an unfilled epoxy [36]. In addition, Ye et al. demonstrated that, with 2.3 wt.% HNT, the impact strength of the PNC is increased four-fold [37]. Lastly, HNTs can be incorporated without requiring intercalation or exfoliation into an epoxy matrix [7]. As a result of the growing interest and promising properties, pristine HNT was used as a nanofiller for this investigation. However, HNT has been shown in epoxies to form isolated clusters or agglomerates that can reach sizes up to several microns and cause deterioration in some mechanical properties [40]. This results from their external surface being largely composed of SiO_2_ groups. Therefore, as the epoxy and the hydroxyl groups of DGEBA are inert towards aluminols and siloxanes under the curing conditions, the interfacial interaction between HNTs and the matrix can be considered as weak [41]. Various techniques have been used to break up these clusters including ball mill homogenization, ultrasonification and surface modification [7,42]. For the latter, it has been demonstrated that surface modification of the nanofillers improves its interactions with the polymer matrix and hence the properties of the nanocomposites [43,44]. For instance, surface modification using polydopamine (PDA), completed through a simple single-step technique, can be applied onto a wide range of inorganic and organic materials [45]. Dopamine is a neurotransmitter found in the human body and can self-polymerize on the surface of a material to form PDA. This leads to a formation of a nanoscale thin film that can be additionally modified for specific applications [46]. Here, dopamine was used to form a thin layer on the surface of HNT. It provided large quantities of hydroxyl groups to support the interfacial bonding and force a better dispersion of the nanofiller in the epoxy matrix [44]. Ultrafine iron trihydroxide (Fe(OH)_3_) nanoparticles (size < 4 nm) were used to endow the PDA-coated HNT nanofillers with improved fire retardancy compared to unmodified HNT. Fe(OH)_3_ nanoparticles have enhanced catalytic abilities due to the exposure of the iron atoms, resulting in a significant increase in both char yield and maximum degradation rate [44]. Although Fe(OH)_3_ is linked to HNT, the nanocomposite with the modified HNT can be considered as a system that contains two different nanoparticles.

The methodology used to study both epoxy-based nanocomposite systems include a combination of conventional differential scanning calorimetry, fast scanning calorimetry, specific heat spectroscopy (SHS) in the form of temperature modulated DSC and FSC (TMFSC) and broadband dielectric spectroscopy. These techniques are used to obtain information about the structure, vitrification kinetics and molecular mobility of the unmodified and surface modified system. In addition, the dispersion and dimensions of the nanofiller were acquired from X-ray scattering and supplemented by transmission electron microscopy (TEM). The data obtained for both systems were directly compared to each other to reveal the effect of the modification on the dispersion and interphase formation. Up to this time, no studies are available on an epoxy-based PNC reinforced with HNT or modified HNT (m-HNT) as a filler using these techniques. To the best of our knowledge, until now, the molecular mobility and interfacial properties have not been discussed for the considered materials.

## 2. Materials and Methods

### 2.1. Materials

The investigated systems are based on bisphenol-A-diglycidyl ether (DGEBA, Epoxydharz C, Faserverbundwerkstoffe Composite Technology, Waldenbuch, Germany) cured with diethylenetriamine (DETA, Sigma-Aldrich, St. Louis, MO, USA). Halloysite nanotubes (HNT, Benahadux Company, Almeria, Spain) were used as the nanofiller. Iron trichloride (FeCl_3_), dopamine hydrochloride (DA-HCl) and urea were obtained from Sigma-Aldrich. The sample preparation is shortly summarized here but described in detail by Li et al. [36]. For chemical details, the reader is referred to the work in [36].

**Figure 1:**
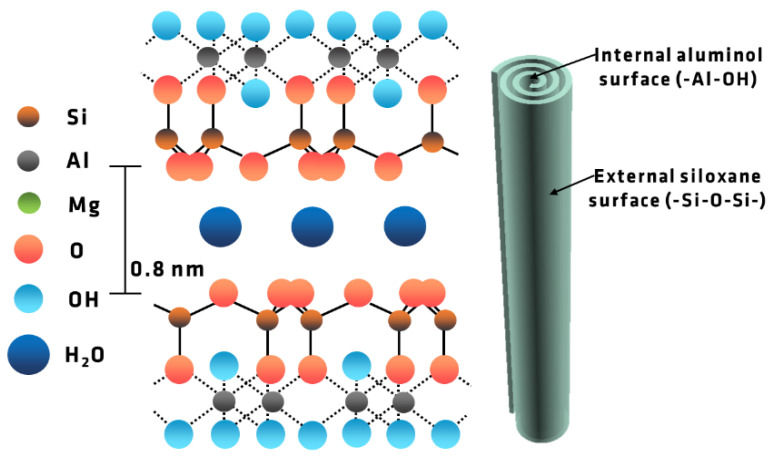
HNT structure with the layers and chemical structure detailed for HNT. Adapted from ref [48].

HNT is an aluminosilicate with the chemical formula Al_2_Si_2_O_5_(OH)_4_·nH_2_O, where, when *n* = 2, HNT is normally in a hydrated state with a layer of water molecules in the interlayer space [44]. HNT has a hollow tubular structure with the outer surface chemically similar to the SiO_2_ [41,47]. The design and chemical structure of HNT is given in Figure 1, where the exterior surface contains siloxane groups and the interior surface has aluminol groups. The length of the tubes ranges from 200 nm to 2 μm, as reported in [44] and addressed below.

The interfacial bonding of the exterior hydroxyl groups of unmodified HNT was found to be insufficient to create a good connection to the epoxy and, hence, needed to be improved. Therefore, dopamine (DA) was used to form a nanocoating layer of polydopamine which has also been shown to increase dispersion of the filler in the matrix [41]. Moreover, the hydroxyl groups of PDA allow for better interactions with the matrix by forming strong hydrogen bonds with the epoxide groups of DGEBA. The modified HNT nanofillers were prepared by first coating them with DA by adding it into a buffer solution (tris(hydroxymethyl)aminomethane, concentrated hydrochloric acid and deionized water) containing the HNT for 24 h at ambient temperature. The obtained product (HNT@PDA) was decorated with ultrafine Fe(OH)_3_ nanodots (size < 4 nm) via a urea-assisted approach. The final system was called HNT@PDA@Fe(OH)_3_ or m-HNT.

Prior to the preparation of the composites, the HNT was dried at 353 K to remove adsorbed water. Unmodified and modified PNCs with varying concentrations were prepared by mixing the epoxy resin with selected amounts of the nanofiller. Next, DETA was added under vigorous stirring. The systems were then cast into a mold and cured for 24 h at 298 K. Lastly, the temperature was increased to 393 K and deep cured for 2 h. The resulting samples were named as Ep/HNTX and Ep/m-HNTX, where X represents the nominal weight concentration of HNT or m-HNT. For the pure Ep sample, the same curing condition was used as stated above.

### 2.2. Methods

#### 2.2.1. Transmission Electron Microscopy (TEM)

TEM measurements were performed using an FEI TECNAI G2 transmission electron microscope (FEI Corporation, Hillsboro, OR, USA) in bright field mode with an acceleration voltage of 200 kV. Samples were prepared by cutting ultrathin slices (70 nm) using a cryo Leica Ultracut UCT ultramicrotome (Leica Microsystems GmbH, Wetzlar, Germany) with a Diatome diamond knife (Diatome Ltd., Bienne, Switzerland).

#### 2.2.2. Small- and Wide-Angle X-ray Scattering (SAXS and WAXS)

The SAXS/WAXS measurements were carried out using MOUSE (Methodology Optimization for Ultrafine Structure Exploration). The X-rays were generated from monochromatized X-ray tubes with Cu Kα (λ = 0.154 nm) and Mo Kα (λ = 0.071 nm) (Xenocs, Grenoble, France). By utilizing two different X-ray sources, it was possible to avoid difficulties with fluorescence from the iron of the m-HNT samples. Data collection was performed using an in-vacuum Eiger 1 M detector (Dectris, Baden-Daettwil, Switzerland), which was placed at multiple distances between 138 and 2507 mm from the sample. The resulting data were processed and scaled to absolute intensity values using the DAWN software package according to standardized procedures considering the propagation of errors [49,50]. Pure epoxy, HNT, m-HNT and PNC systems (Ep6/HNT, Ep12/HNT, Ep15/HNT, Ep6/m-HNT and Ep15/m-HNT) were investigated employing this technique.

#### 2.2.3. WAXS: Linear Combination of the Data for the Pure Epoxy and the Nanofiller

As the SAXS/WAXS data were scaled to absolute intensity, it was possible to perform a linear combination of Ep and HNT/m-HNT WAXS data to obtain a rough estimate of the volume fractions for both components [11,27]. For the HNT and m-HNT samples, data were collected using two different X-ray sources (Xenocs, Grenoble, France), in multiple sample positions, with no observed change in the ratio of peak intensities, indicating that the measured samples lack orientation, and a decent powder diffraction average signal was collected. This also remained true for the PNC samples with HNT and m-HNT particles embedded within the epoxy. Hence, a linear combination was performed along a q-range of 6–50 nm^−1^. From the obtained scaling factors (from a least-squares optimization), a rough volume fraction was determined for both the epoxy and the HNT/m-HNT present in PNCs. For this analysis, epoxy, HNT and m-HNT were assumed to have atomic compositions of C_47_H_56_O_9_N (1.13 g cm^−3^), Al_2_H_2_O_9_Si (2.4 g cm^−3^) and Al_2_H_5_O_12_SiFe (2.708 g cm^−3^), respectively.

#### 2.2.4. SAXS Pattern Simulations from 3D Objects

SPONGE, a scattering pattern calculator written in Python (Python Software Foundation, Wilmington, DE, USA), was utilized to verify features observed in the SAXS patterns from simulations of rolled cylinders [51,52,53]. SPONGE can calculate scattering patterns from three-dimensional objects, with the ability to add polydispersity and structural dynamics to the system, while staying as close as possible to the first principles of scattering. It utilized the Debye equation to calculate the scattering patterns from randomly distributed, infinitesimally small points, randomly distributed inside of the object surface [54,55].

For this analysis, three-dimensional rolled cylinders were generated in OpenSCAD and exported to STL files (STereo Lithography) [56]. Different models were generated to show how the different structural parameters affect the simulated SAXS patterns. Changes to the length (D_length_), outer diameter (D_outer_), Lumen diameter (number of rolls) (D_lumen_) and thickness of the rolled sheet (D_sheet_) were probed, as well as polydispersity (σ) (Gaussian size distribution). All parameters were kept constant between simulations except the specific parameter being changed. As a base model, the following parameters were used: length = 100 nm, outer diameter = 13 nm, lumen diameter = 5 nm and thickness = 1 nm. This base model was also used to investigate polydispersity with a Gaussian distribution, with values of the standard deviation (σ) between 0.1 and 0.5. In general, the SPONGE simulations were performed with 1000 points inside the rolled cylinders, 200 points logarithmically divided along the q-vector within 0.018–20 nm^−1^ and 500 independent repetitions of the simulation.

#### 2.2.5. Differential Scanning Calorimetry (DSC)

The conventional DSC measurements were performed by a power compensated Perkin Elmer DSC 8500 instrument (Perkin Elmer, Waltham, MA, USA) with a heating/cooling rate of 10 K min^−1^ in the temperature range of 298–493 K. Samples with masses of ca. 8.5 ± 0.2 mg were placed in 50 µL aluminum pans. The data of the second heating run were taken for analysis to allow the samples to be completely cured for a detailed comparison of the results. Nitrogen was used as the protection gas at a flow rate of 20 mL min^−1^. Baseline corrections were performed by measuring empty pans where this data was subtracted from that of the samples. Temperature and heat flow calibration was performed using indium as a standard. Each sample was measured approximately 3 times and the error in *T_g_* was below 2 K.

#### 2.2.6. Temperature Modulated DSC (TMDSC)

TMDSC was carried out using a Perkin Elmer DSC 8500 device (Perkin Elmer, Waltham, MA, USA), employing the StepScan^®^ approach, where alternating steps of heating and isothermal times were applied, resulting in a periodic heat flow. A heating rate of 60 K min^−1^ with a step height of 2 K was used. The length of the isothermal steps resulted in frequencies between 8.3 × 10^−3^ and 3.3 × 10^−2^ Hz (isotherm times from 120 to 30 s). Nitrogen was used as a purge gas at a flow rate of 20 mL min^−1^. The complex specific heat capacity (c_p_*) was estimated as the area under the heat flow peak divided by the height of the predefined temperature step. Absolute values of the heat capacity were obtained by synthetic sapphire as reference material. The modulus of complex specific heat capacity $|c_{p}^{*}|=\sqrt{c_{p}^{\prime 2}+c_{p}^{\prime \prime 2}}$ (the so-called reversing heat capacity) was considered, where $c_{p}^{\prime }{\;\mathrm{and}\;c}_{p}^{\prime \prime }$ are the real and imaginary parts of $c_{p}^{*}$.

#### 2.2.7. Fast Scanning Calorimetry (FSC)

FSC was used to extend the vitrification investigations to higher heating rates. A Mettler Toledo Flash DSC 1 (Mettler Toledo, Greifensee, Switzerland), a chip-based power compensated DSC, was used in conjunction with micro electrical mechanical systems (MEMS) sensors to allow heating rates up to 4 × 10^4^ K^−1^. For each sample, a specific non-adiabatic chip sensor was used with an active reference and the sample site. The samples were prepared by cutting 10 μm thick pieces using a cryo-microtome. Since thermosetting materials are unable to be melted, the high viscosity silicon oil AK 60,000 from Wacker Chemie AG (Burghausen, Germany) was used to improve the thermal contact between the sample and sensor and to reduce the thermal lag. Silicon oil was placed on both the sample and reference sensors to account for its additional heat capacity in the measurements. For the investigations of the glass transition, the data from cooling is commonly used. However, the thermal lag was insignificant between heating and cooling due to the small sample dimensions. Therefore, here the data of the heating run were used. Nitrogen was used as a purge gas at a flow rate of 40 mL min^−1^.

#### 2.2.8. Temperature Modulated FSC (TMFSC)

This technique is based on the Mettler Toledo Flash DSC 1. Similar to TMDSC, a modulated temperature profile was applied by heating and isothermal steps. The complex heat capacity ($C_{p}^{*}$) was obtained as Fourier transformation of the instantaneous heat flow (*HF*) over the Fourier transform of the heating rate ($\dot{T}$).

(1) $$C_{p}^{*}=\frac{\int_{0}^{t_{p}}HF\left(t\right)e^{-i\omega t}dt}{\int_{0}^{t_{p}}\dot{T}\left(t\right)e^{-i\omega t}dt}$$

where 𝜔 is the angular frequency. For these investigations a step height of 2 K with heating rates of 200 and 2000 K/s were used with isothermal times *t_p_* = 1, 0.1 and 0.05 s. The frequency ($f=\frac{2\pi }{\omega }$) was noted as the inverse of each isothermal time. The sparse fast Fourier transformation (SFFT) was employed, which gives also higher harmonic frequencies (from 1 to 11 Hz for 1 s, from 10 to 110 Hz for 0.1 s and from 20 to 220 Hz for 0.05 s). Again, the modulus of the complex heat capacity was used for analysis.

#### 2.2.9. Broadband Dielectric Spectroscopy (BDS)

BDS was used to investigate the molecular mobility of the materials in the frequency range from 10^−1^ to 10^6^ Hz [15,57]. A periodic electric field with a small field strength was applied as an input and the time-dependent polarization was measured as output. The polarization arises from the orientation of permanent dipoles in the material and can be quantified using the complex dielectric function ε*(ω) = ε’(ω) – 𝑖ε″(ω) where ε′(ω) is the real (storage) part of the complex dielectric function and ε″(ω) is the imaginary (loss) part. The dielectric properties of the samples were measured in the temperature range from 173 to 503 K. BDS was carried out by a Novocontrol high-resolution ALPHA analyzer (Novocontrol Technologies, Montabaur, Germany) connected to an active sample cell. A Quatro Novocontrol cryosystem employing a temperature stability better than 0.1 K was used for temperature control. All samples were placed between two gold plated electrodes (Ø = 20 mm) in a parallel plate geometry. Gold electrodes were evaporated onto the surfaces of the samples in an ultrahigh vacuum (10^−5^ mbar) to establish a good electrical contact. The impedance Z*(ω) was measured and the complex dielectric function was calculated from the impedance.

## 3. Results and Discussion

### 3.1. Epoxy Nanocomposite with Halloysite Nanotubes (Ep/HNT)

#### 3.1.1. X-ray Scattering

**Figure 2:**
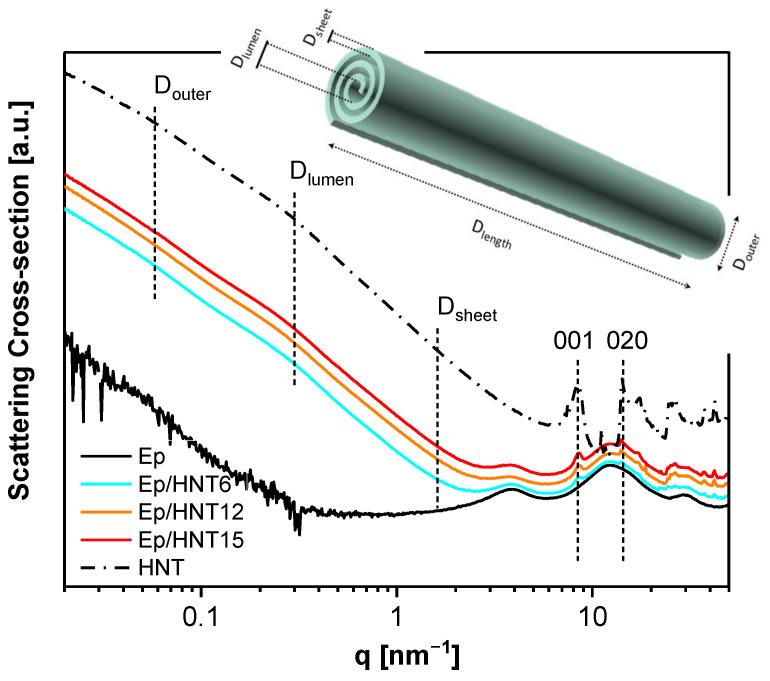
X-ray scattering patterns of the pure epoxy, halloysite nanotubes and nanocomposites as indicated. The curves are shifted along the *Y*-axis for the sake of clarity. The raw scattering data at absolute values of the scattering cross-section are shown in Figure S2. The inset gives a scheme of the halloysite rolled cylinder with different dimensions indicated.

To study the structure and morphology, X-ray scattering experiments were performed on pure epoxy, HNT and three nanocomposites (Ep/HNT6, Ep/HNT12 and Ep/HNT15). The scattering patterns are shown in Figure 2. The (001) peaks found for pure HNT (8.42 nm^−1^) indicate layer spacings of 0.75 nm. However, these peaks are relatively broad indicating that there is distribution in the observed layer spacing, ranging between ca. 0.63 and 0.93 nm. The presence of the (020) reflection at 14.2 nm^−1^ indicates that the HNT sample is present in a dehydrated state and tubular in shape [58,59].

The scattering pattern of the pure epoxy shows three distinct scattering features in the WAXS region, located at 3.88, 12.46 and 29.8 nm^−1^. These broad bumps in the X-ray scattering patterns of amorphous materials with no long-range correlations can be taken as a fingerprint for short-range correlations within the epoxy [60]. A detailed analysis of the origin of the three-peak structure for an anhydride epoxy material can be found in [27], where such an X-ray scattering was related to an inherent spatial heterogeneity of the crosslinked network. The intermediate bump with the highest intensity, at q = 12.46 nm^−1^, is the amorphous halo, corresponding to the most frequently occurring intermolecular distances. The peak at q = 3.88 nm^−1^ was assigned to isopropylidene groups between the phenyl rings forming elongated clusters with an average size of ca. 1.6 nm, in the Bragg approximation. The peak at q = 29.8 nm^−1^ (averaged intermolecular distances of ca. 0.21 nm) can be correlated with regions in the network with lower crosslinking density than the average, as discussed in [11].

For the composites, the position of the peaks related to the crystal structure of HNT remain unaffected, indicating that the nanotubes maintained approximately their structure upon incorporation in the matrix. Moreover, the amorphous halo and the two other broad features in the X-ray scattering are also found at the same positions as that for the pure epoxy. Therefore, it is concluded that X-ray investigations did not detect large differences in the structure of the matrix in the PNCs compared to the pure epoxy.

**Figure 3:**
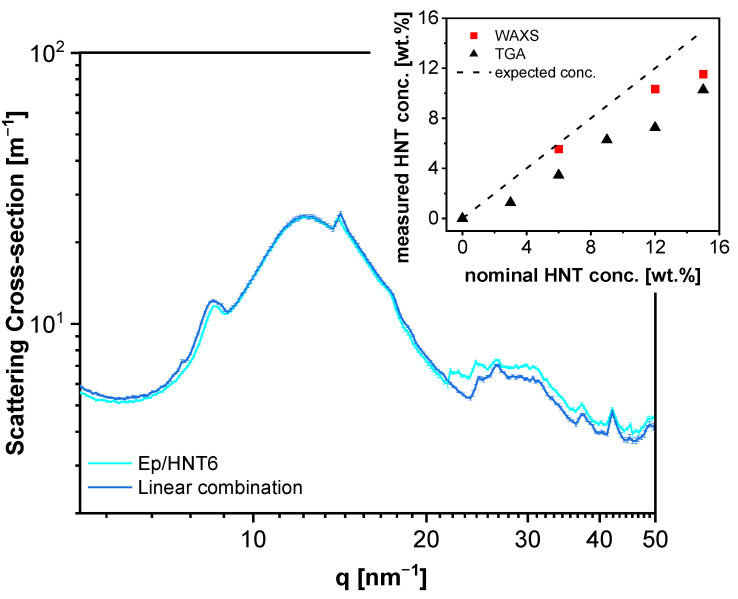
Analysis of the X-ray data for Ep/HNT6 in the WAXS region by fitting a linear combination of the pure epoxy and HNT nanotubes data. Experimental data, light blue; fit function, dark blue. The inset represents the weight fractions of the nanofiller estimated by X-ray scattering and thermogravimetric analysis as a function of the HNT nominal concentration. The dashed line represents the one-to-one relationship.

An example for the analysis of the WAXS data by the linear combination is shown in Figure 3. This analysis, carried out for Ep/HNT6, 12 and 15, revealed that the concentration of the HNT is lower than the nominal one (inset Figure 3). The calculated values from the WAXS data agree with the weight fractions obtained from thermogravimetric analysis (TGA, for details see the Supplementary Materials). This means both methods prove that the HNT concentration in the composites is lower than formulated.

**Figure 4:**
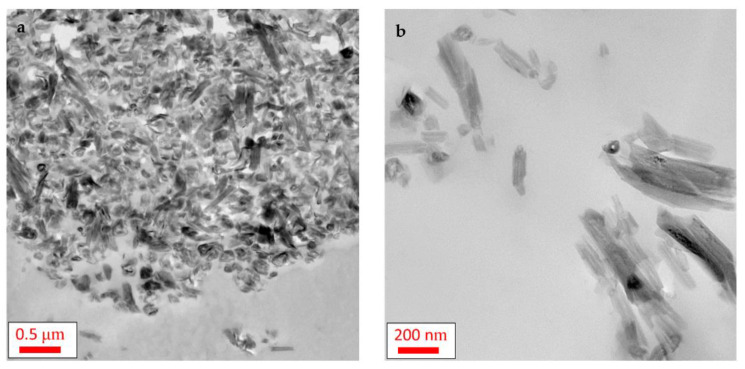
TEM images for Ep/HNT15: (**a**) lower magnification; and (**b**) higher magnification.

The SAXS data for HNT and the PNCs were analyzed using McSAS, a Monte Carlo fitting method for extracting form-free size distributions [50] (see Figure S3). From this analysis, three distinct size regimes were observed in the (volume-weighted) distributions, at ca. 4, 19 and 125 nm. To assign these values to the complex morphology of HNT, simulations of SAXS profiles from 3D models were performed. The simulated SAXS curves from 3D models with varying structural parameters can be seen in Figure S4. For these simulations, only a single parameter was altered each time to observe how each structural feature can affect the simulated SAXS profiles. Thus, it was possible to assign certain features of the SAXS data to different features in the rolled cylinders. Three features are observed in the SAXS data, which are representative of D_outer_, D_lumen_ and D_sheet_, with D_length_ > 400 nm (inset Figure 3) being greater than that of the observable experimental SAXS range. It was found that the HNT within the PNCs has average dimensional properties of D_outer_ = ~125 nm, D_lumen_ = ~19 nm and D_sheet_ = ~4 nm. It was found that D_outer_ and D_lumen_ are different for the nanocomposites and the pure HNT. D_outer_ increases for the nanocomposites (~125 nm) by approximately 18 nm, compared to pure HNT (~ 107 nm), where D_lumen_ for the PNCs is ca. 19.2 nm, whereas for HNT it is ca. 19.7 nm. These changes in the outer and lumen diameter of the nanofiller embedded in the matrix, in comparison to the pure HNT, might be discussed in terms of a swelling of the NPs due to the entering of small molecules into the rolls. Considering that HNT is added to the reaction mixture before the crosslinking reaction it can be assumed that one or both components (DGEBA and/or DETA) enter the rolls of the tube, widening the overall diameter and decreasing the inside space of the tubular structure. Nevertheless, the distribution of D_outer_ values is quite broad, thus it cannot be excluded that larger D_outer_ is observed for PNCs than the pure filler due to a smoother electron density transition, leading to a greater power-law approximation in the simulations. These dimensions are complimentary to those seen by transmission electron microscopy (Figure 4) and those given in the literature [52,53]. To further confirm the observed features, simulations of larger rolled cylinders that more closely represent these parameters were undertaken. From these simulations, a good correlation between D_outer_ and D_lumen_ was observed. However, due to difficulties in the simulation of data at high q values and the implementation of a global polydispersity across the whole model (Gaussian distribution) in SPONGE, rather than the separation of polydispersity parameters across the different parameters as would be expected from real samples, correlating features towards higher-q becomes difficult to observe.

#### 3.1.2. TEM

The morphology of the system was further investigated using transmission electron microscopy. Figure 4 shows the TEM images obtained for Ep/HNT15 at two different positions of the sample at two magnifications. Figure 4a, taken with a lower magnification, shows that HNTs are dispersed in agglomerates in the epoxy matrix, forming clusters on the length scale of some μm. For other regions, depicted in Figure 4b, the HNTs are dispersed more or less separately in the matrix. Moreover, the shape of the HNT is unaltered, maintaining the tubular shape when embedded in the matrix as also confirmed by the X-ray measurements. Additional TEM images were obtained for the pure epoxy and Ep/HNT6 (Figure S5).

#### 3.1.3. Calorimetry

**Figure 5:**
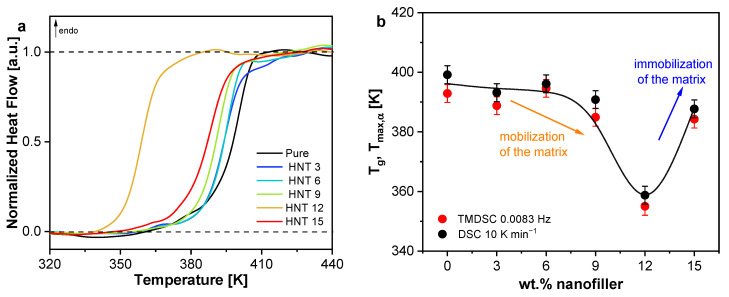
(**a**) Normalized heat flow curves as described in the text for the indicated materials. (**b**) Glass transition temperature as a function of the nominal filler content for conventional DSC and TMDSC, estimated as described in the text. Error bars are given and a line as a guide for the eyes for the DSC data.

The thermal properties of the pure epoxy and the unmodified PNCs (Ep/HNT3–Ep/HNT15) were first investigated by conventional DSC. The normalized heat flow curves ($HF_{norm}=\frac{HF-HF_{glass}}{HF_{liquid-HF_{glass}}})$ are shown in Figure 5a. The second heating run was taken for analysis for the reasons discussed above. The glass transition temperature (*T_g_*) was estimated by fitting a sigmoidal function to the heat flow curve measured at 10 K min^−1^. Next, the derivative of the fit was taken with respect to temperature (Figure S6). A Gaussian was further fitted to the resulting peak and the maximum position of the Gaussian was taken as *T_g_*. Figure 5b depicts the *T_g_* values from DSC as a function of the nominal filler content for the heating rates of 10 K min^−1^.

The glass transition temperature decreases compared to the unfilled sample with increasing nominal nanofiller concentration till 12 wt.%, followed by an increase of *T_g_* for 15 wt.% (see Figure 5b). The complex concentration dependence of *T_g_* was observed for all employed thermal methods. It can be discussed to arise from two competing mechanisms: (1) a mobilization; and (2) an immobilization of segments of the epoxy matrix due to the nanofiller.

(1) The mobilization is related to a decrease in the crosslinking density of the epoxy due to the nanofillers, which are acting as spatial obstacles and affecting the diffusion-controlled curing reaction [27]. Hence, for all PNC concentrations, a reduction in crosslinking density should take place, and, thus, the glass transition temperature decreases compared to the unfilled epoxy. Another approach to discuss the reduced crosslinking density was a preferential adsorption and/or the intercalation of one reactant onto the nanofillers. Some amount of this reactant would then be unavailable for the curing reaction and also result in a lower crosslinking density. It should be noted that an intercalation of one of the reaction partners was suggested by the X-ray measurements. Furthermore, other scenarios of the mobilization of an epoxy-matrix by SiO_2_-based nanofillers are discussed in the literature. Sun et al. investigated the effect of nanofillers on glass transition behavior for various epoxy nanocomposites and showed that for silica particles there was a significant decrease of around 30 K for the nanocomposites compared to the unfilled epoxy, citing residual organics and bonded water assisting molecular motions and creating additional free volume at the resin-filler interface [61]. Piscitelli et al. [20] showed a large reduction (~25 K) in *T_g_* for an epoxy PNC with silica nanoparticles. The reduction in *T_g_* was observed for 3 wt.% SiO_2_ and was attributed to a plasticizing effect [62]. Piscitelli assigned the plasticizing effect to arise from the siloxane component entering the epoxy network and forming epoxy-siloxane sequences [20], thereby increasing the distance between the epoxy units. Relating this to HNT, where siloxane groups are present on the surface of the nanofillers, the depression of 30 K seen at 12 wt.% can be assigned to a plasticizing effect as well. Here, the HNT surface is similar but not identical to that of SiO_2_ discussed in [20]. Therefore, the strong reduction of the *T_g_* value observed for Ep/HNT12 cannot be unambiguously assigned to the plasticizing effect of the external siloxane groups, although it is possible that such a mechanism can contribute to the decrease of *T_g_*. Another important factor is the large aspect ratio of tubular nanofillers, which can significantly affect the curing reaction and thus the crosslinking density, especially for higher NPs concentrations.

(2) The second mechanism, the immobilization of segments due to the nanofiller, can be discussed in the frame of two scenarios. On the one hand, an immobilized fraction can be formed, the rigid amorphous fraction, where polymer segments are adsorbed on the surface of the nanofiller and therefore do not contribute to the cooperative segmental mobility increasing the glass transition temperature values. On the other hand, for a tubular nanofiller with a high aspect ratio, a percolation effect should be considered. A bridged network between the nanofiller and epoxy matrix can be formed for higher nanofiller concentrations, which restricts the segmental fluctuations of the system resulting in an increase in *T_g_.* Ye et al. [37] reported that, for 2.3 wt.% HNT in an epoxy, the dispersion of HNT resulted in a two-phase structure. One phase consisted of HNT nanotubes homogenously dispersed with large inter-tube distances, forming an epoxy-rich region. In the second phase some of the halloysite nanotubes were dispersed with a short inter-tube distance resulting in an HNT-rich region. TEM images revealed that the spaces among the HNTs in this region were filled with epoxy. Ye et al. referred to this region as high content HNT embedded in a continuous matrix phase. For higher HNT concentration, this effect might become more pronounced, as more HNT are embedded in the continuous phase. The epoxy-matrix in this region will be exposed to additional confinement effects due to the short inter-tube distances. This assumption is confirmed by the TEM image for Ep/HNT15, given in Figure 4a, where the nanofillers are clustered together with some epoxy in between. Due to the additional confinement in this HNT-rich region, the polymer segments might be restricted in the cooperative segmental mobility, increasing *T_g_*. Considering that the increase of *T_g_* is found for the highest concentration of HNT, Ep/HNT15, this can be considered a critical concentration, where additional confinement in the HNT-rich regions of the PNC affects the overall segmental mobility of the whole matrix.

#### 3.1.4. Specific Heat Spectroscopy

In addition to the information about the vitrification of the system, the segmental dynamics can be revealed using TMDSC. Specific heat capacity is a measure of the segmental mobility related to the glass transition of the samples. The modulus of the complex specific heat shows a step at the glass transition, similar to the heat flow curves from DSC. The step in the heat capacity was analyzed using the sigmoidal fit and derivative method to obtain the frequency dependent glass transition temperatures *T_max,α_*. As shown in Figure 5b as an example, the composition dependence of *T_max,α_* at f = 0.0083 Hz has the same dependence as the *T_g_* obtained from the two static techniques, further confirming the complex concentration dependence of the glass transition temperature for this system.

##### Interfacial Properties

Moreover, the modulus of the complex specific heat can be used to quantify both the mobile and rigid amorphous fractions with respect to the cooperative segmental motions. This analysis is based on the estimation of the step height of the specific heat capacity at the glass transition, denoted as the calorimetric strength Δc_p_. For a PNC, Δc_p_ is expected to decrease compared to the unfilled material in the case that an immobilized interface is formed. In the three-phase model of a PNC, an amorphous nanocomposite is composed of mobile fraction (MAF), a possible RAF and the nanofiller. It should be noted that this concept was initially introduced by Wunderlich for semicrystalline polymers and further extended to polymer-based nanocomposites [63]. MAF and RAF can be calculated by

(2a) $$MAF=\frac{\Delta c_{p,PNC}}{\Delta c_{p,\;pure\;epoxy}}$$

(2b) RAF=1−filler content−MAF

For epoxy-based nanocomposites, mobilization and immobilization effects can occur simultaneously, as discussed in [16], where the calorimetric strength was found to initially increase, reaching values higher for the nanocomposite than for the pure epoxy, followed by a subsequent decrease of Δc_p_.

**Figure 6:**
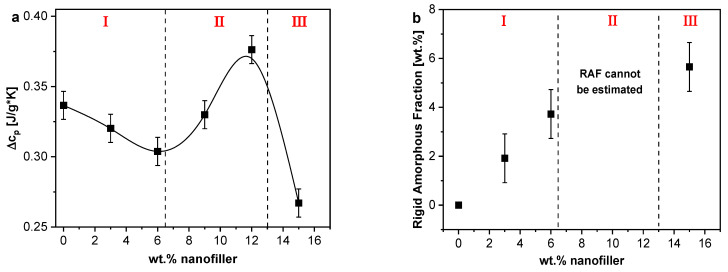
(**a**) The calorimetric strength, Δc_p_; and (**b**) the calculated RAF as a function of filler content. Error bars are included. In (**a**), the line is a guide to the eyes.

Figure 6a depicts the calorimetric strength at f = 0.0083 Hz as function of the nominal HNT concentration. The observed dependence can be divided in three different parts. In the first part (I) for concentrations between 0 and 6 wt.% HNT, Δc_p_ decreases with increasing filler concentration. This decrease means that the number of mobile segments decreases and thus the dominating effect is a formation of RAF. The amount of RAF increases with increasing concentration, as shown in Figure 6b, reaching up to 4% wt.% RAF for 6 wt.% HNT. 

In the next part (II) between 6 and 12 wt.% of HNT, an increase in Δc_p_ is observed reaching values higher than those observed for the unfilled epoxy. This means the dominating effect is a mobilization and hence the number of mobile segments is increased in comparison to unfilled epoxy. This can be attributed to a filler-related decrease in the crosslinking density as discussed above. This is in agreement with the decrease of *T_g_* observed for 12 wt.% HNT. For all PNC samples, an immobilization and mobilization take place simultaneously; however, through TMDSC, the dominating effect is accessible. Therefore, in the first part (I), the decrease in the crosslinking density also occurs, but it is dominated by the formation of RAF. In the second part (II), the formation of RAF probably also takes place but is strongly overlaid by an increase of the number of mobile segments in the matrix. In contrast to previous observations, for flake-shaped Boehmite nanoparticles (BNP) in an anhydride-cured DGEBA epoxy matrix [27], where the increase in the number of mobile segments was observed for 1 wt.% of BNP followed by a consistent decrease of Δc_p_ with increasing BNP content, indicating an immobilization of segments. Here, the system under investigation is reinforced with nanofillers with different dimensions. The nanofiller has the form of a nanotube with a length > 400 nm compared to BNP with a primary diameter of ~14 nm. Consequently, the nanotubes impose more restrictions to the crosslinking reaction at higher concentrations compared to other shaped NPs discussed in the literature [64]. If one tried to calculate RAF for HNT concentrations between 6 and 12 wt.% HNT, negative values would be obtained. Since negative values of RAF contradicts its definition, this consideration indicates that the formation of RAF is strongly overlaid by the creation of mobile segments due to a decrease in crosslinking density. In other words, the latter process strongly dominates over the formation of RAF.

In the last part (III) for HNT concentrations between 12 and 15 wt.%, a sharp decrease of Δc_p_ is observed. Hence, the formation of RAF becomes again the dominant effect, which results in an effective value of RAF about 5 wt.%. As suggested by the increase of *T_g_* in this concentration region, an additional reason for the increase of RAF might be related to the existence of an interconnected pathway of the polymer segments (percolating network). This behavior becomes more relevant at this nanofiller concentration [65], influencing the overall number of mobile segments by introducing additional confinements to the segmental fluctuations. This additional confinement might be due to the bridged network formed between the polymer and the nanofiller in the HNT-rich region.

**Figure 7:**
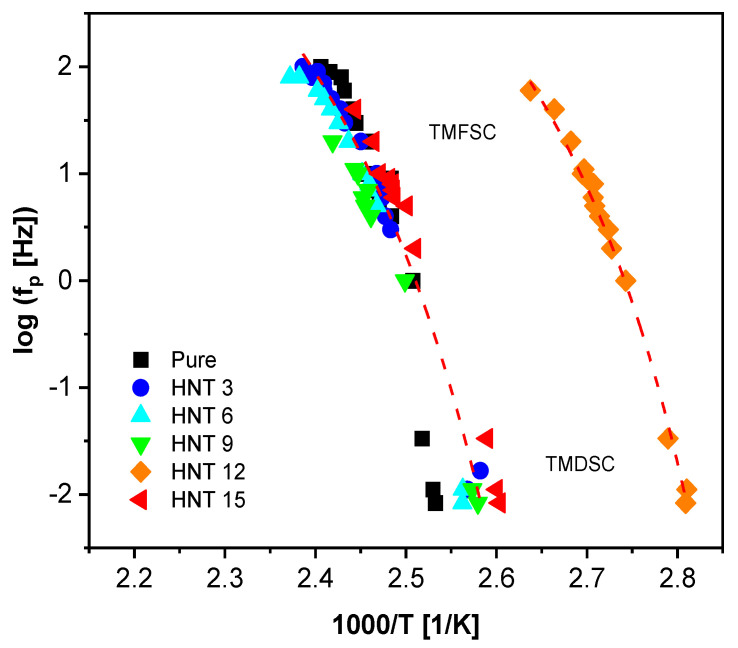
Combined calorimetric activation plot for TMFSC and TMDSC: black squares, pure epoxy; blue circles, Ep/HNT3; teal triangles, Ep/HNT6; green triangles, Ep/HNT9; orange diamonds, Ep/HNT12; red triangles, Ep/HNT15. The red lines are exemplary VFT fits to the data of Ep/HNT3 and Ep/HNT12.

In addition, temperature modulated fast scanning calorimetry was carried out to extend the investigation of the segmental dynamics by calorimetry in a broader frequency and temperature range. The combined calorimetric activation plot, where the logarithm of the relaxation rate is plotted as a function of inverse temperature, is depicted in Figure 7. The temperature dependence of the relaxation rates is curved in such a representation as expected for the dynamic glass transition. The Vogel–Fulcher–Tammann [66,67,68] (VFT) equation was employed to describe the temperature dependence of the relaxation rates. The VFT equation is given by

(3) $${ƒ}_{p,\alpha }\left(T\right)=\frac{1}{2\pi \tau \left(T\right)}={ƒ}_{\infty }\exp\left(\frac{-DT_{0}}{T-T_{0}}\right).$$

${ƒ}_{\infty }$ is a pre-exponential factor (${ƒ}_{\infty }\approx$ 10^10^–10^12^ Hz); *T*_0_ is the Vogel temperature or ideal glass transition temperature, which is found 20–40 K below the thermal glass transition temperature; and *D* is the fragility parameter that is used to quantify glass forming systems. The VFT fit lines are included in Figure 7, and the data obtained from the two frequency dependent techniques can be described by the same fits. The temperature modulated calorimetric relaxation data are further discussed when compared to the results obtained from BDS.

#### 3.1.5. Broadband Dielectric Spectroscopy

**Figure 8:**
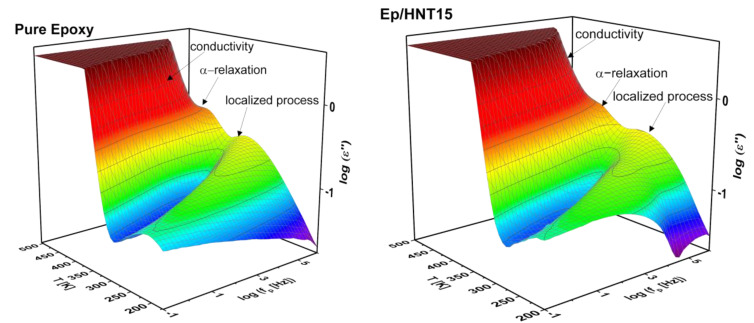
Three-dimensional plots of dielectric loss as a function of frequency and temperature for pure epoxy and the nanocomposite with 15 wt.% HNT.

The dielectric loss as function of frequency and temperature for the pure epoxy and 15 wt.% HNT is depicted as 3D representations in Figure 8. Three dielectric processes are identified in both 3D plots. One process is the segmental relaxation labeled as α-relaxation which is active at temperatures above the glass transition temperature. This process is related to cooperative segmental fluctuations within the epoxy network. It is hidden by a large increase in dielectric loss due to conductivity and polarization effects arising from the migration and/or blocking of charge carriers which are due to remaining ion impurities. This feature is considered as a parasitic effect and is not relevant to the scope of this work [28]. Further, at lower temperatures than relevant for the α-relaxation, a broad relaxation process is found, assigned to the local molecular fluctuations such as the β-relaxation.

**Figure 9:**
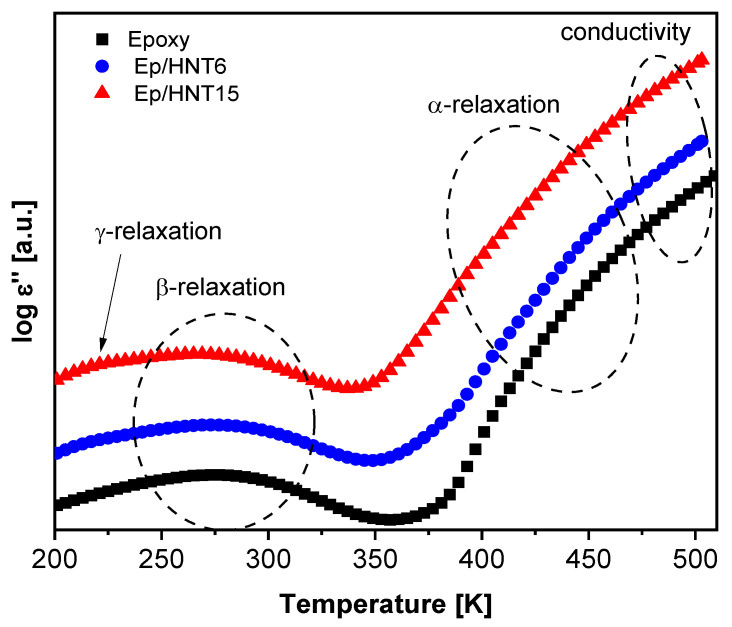
Dielectric loss spectra at a frequency of 1000 Hz for selected samples. Black squares, unfilled epoxy; blue circles, Ep/HNT6; red triangles, Ep/HNT15. The dielectric loss is shifted along the *y*-axis for sake of clarity.

For a more detailed discussion of the dielectric behavior, the dielectric loss is plotted in Figure 9 as a function of temperature (isochronal representation) at f = 10^3^ Hz for the pure epoxy and Ep/HNT6 and Ep/HNT15. Similar to the 3D plots, three well pronounced dielectrically active features can be identified. However, in Figure 9, an additional low-temperature relaxation process can be observed as a low intensity shoulder of the β-relaxation, denoted as γ-relaxation.

##### Localized Molecular Fluctuations

**Figure 10:**
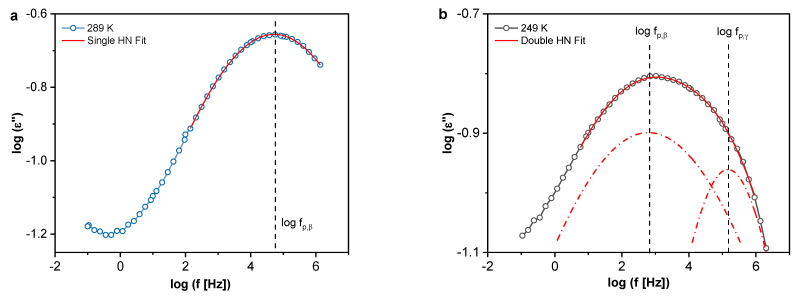
(**a**) Single HN function fit; and (**b**) double HN function fit with the labeled relaxation rates for Ep/HNT3.

Dielectric loss data are commonly analyzed in the frequency domain (isothermal) by fitting the Havriliak–Negami (*HN*) function [69,70] to the dielectric loss plots. The HN function reads

(4) $$\varepsilon_{HN}^{*}\left(\omega \right)=\varepsilon_{\infty }+\frac{\Delta \varepsilon }{{\left(1+{\left(i\omega \tau_{HN}\right)}^{\beta }\right)}^{\gamma }}$$

where Δε is the dielectric strength, $\varepsilon_{\infty }=\underset{\omega \to \infty }{\lim}\varepsilon^{\prime }\left(\omega \right)$ and $\tau_{HN}$ is the characteristic relaxation time. β and γ are fractional shape parameters (0 < β ≤1 and 0 < βγ ≤ 1) that describe the broadening and asymmetry of the peak, respectively. The relaxation rate ($f_{p}$) is then obtained as the frequency of the peak maximum position of the dielectric loss using the obtained fitting parameters. An example of the HN fit to the data for Ep/HNT3 at 298 K is shown in Figure 10a. In accordance with the 3D plots, at 289 K, one dielectrically active process is found, denoted as β-relaxation. The molecular origin of this relaxation has been discussed previously [57,71,72,73], with the β-relaxation being assigned to the crankshaft motions of small fragments of segments between crosslinks [74]. However, in another approach, this relaxation was associated with the localized motions of dipoles formed during the crosslinking reactions (hydroxyl ether and secondary or tertiary amine groups) [75]. Ochi et al. proposed that it was the sum of the contributions from the hydroxyether group for bisphenol-A-type epoxide resins cured with aliphatic amines [72]. It has also been discussed that the β-relaxation arises from the local motions of dipoles of unreacted components during curing, specifically epoxide rings [73]. Recent findings for a similar amine-cured system employing low field proton nuclear magnetic resonance (NMR) spectroscopy [11] reveal that, due to the significant amplitude of proton motions, β-relaxation is probably associated with the rotational motions of para-substituted phenyl rings of DGEBA, i.e., phenyl rings with the adjacent ether linkages.

Furthermore, as previously discussed, at temperatures below approximately 280 K, a second low intensity shoulder emerges at high frequencies compared to the β-relaxation, denoted as γ-relaxation. A sum of two HN functions were then fitted to the data and two characteristic relaxation times were determined. Figure 10b shows the fit of the two HN functions as example and the positions of the β- and γ-relaxations. The γ-relaxation is a low intensity process related to groups with either a weaker dipole moment or a lower number density of fluctuating dipoles involved in this process. In addition, this process is more pronounced for the PNCs than for the pure epoxy, increasing in intensity with increasing HNT content. For higher concentrations of NPs epoxy networks tend to be more heterogeneous, having for instance more “dangling ends” (unreacted end groups of the resin/hardener). Therefore, it can be assumed that the γ-relaxation is related to the local motions of those dipoles that remain as the unreacted components during curing (see Figure 9).

**Figure 11:**
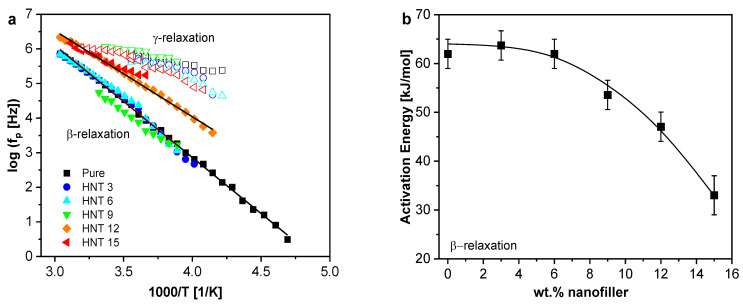
(**a**) Activation plot for the β-relaxation (solid symbols) and γ-relaxation (open symbols). Black squares, pure epoxy; blue circles, Ep/HNT3; teal triangles, Ep/HNT6; green triangles, Ep/HNT9; orange diamonds, Ep/HNT12; red triangles, Ep/HNT15. Black lines are Arrhenius fits to the data. (**b**) The activation energy for the β-relaxation as a function of the HNT concentration.

The logarithm of the relaxation rates as a function of inverse temperature were plotted into a relaxation map, as given in Figure 11a. Both processes for the pure epoxy and the PNCs follow the Arrhenius equation (Equation (5)), as typical for localized processes.

(5) $$\log f_{p}=f_{\infty }\exp\left[\frac{-E_{A}}{RT}\right].$$

Here, $f_{\infty }$ is the relaxation rate at infinite temperatures, *E_A_* is the activation energy and R is the ideal gas constant. As stated above, the γ-relaxation is seen as a low intensity shoulder of the β-relaxation, therefore the dependence of the intensity on the concertation of the nanofiller cannot be quantitatively addressed. Because of the scatter in the relaxation rates, only an averaged activation energy *E_A_* of ca. 28 kJ mol^−1^ can be given. The errors for the activation energy here are below 3 and 4 kJ mol^−1^ for the highest nanofiller concentration. In Figure 11b, the estimated activation energies for the β-relaxation are plotted versus the concentration of HNT. *E_A_* decreases systematically from 65 to 29 kJ mol^−1^ with increasing nanofiller concentration. To discuss this finding, it should be noted that the crosslinking density depends on the nanofiller content and lower crosslinking densities are expected for higher nanofiller contents. Therefore, the free volume in the system might increase, leading to a decrease of the activation energy. The decrease of the activation energy for the PNCs compared to the pure epoxy also confirms the theory suggested by Hassan et al. [75] that the β-relaxation is sensitive to differences in the distribution of local free volume, related to the different crosslinking density. Hassan et al. studied two bisphenol-A based epoxies cured with different crosslinking agents. The position of the β-relaxation peak for both systems differs due to different crosslinking densities of the samples. Additionally, in a free volume study by positron annihilation lifetime spectroscopy (PALS) performed by Patil et al. [76] for bisphenol-A resins cured with different curing agents, the epoxies with a lower crosslinking density had a higher free volume. The activation energies for these epoxies were also estimated where the samples with the higher free volume showed a lower activation energy.

##### Segmental Dynamics

As discussed above, the segmental dynamics (α-relaxation) of the system is hidden by conductivity. Therefore, to minimize this contribution, a conduction-free analysis [77] was carried out. The real part of the complex dielectric function ε′ was differentiated with respect to frequency. For the Debye function, this approach results in: (I) a peak appearing in the derivative of ε′ named $\varepsilon_{deriv}^{\prime \prime }$; (II) due to the square, the peak becomes narrower than the peak observed for ε″ (see Equation (6)); and (III) the Ohmic conductivity contribution (ε″~1/ω) is removed because ε′ is independent of frequency for that case.

(6) $$\varepsilon_{deriv}^{\prime \prime }=-\frac{\partial \varepsilon^{\prime }\left(\omega \right)}{\partial \ln\omega }\;\sim \;{\left(\varepsilon^{\prime \prime }\right)}^{2}$$

For the HN function, the derivative of the real part is calculated as

(7) $$\frac{\partial \varepsilon_{HN}^{\prime }}{\partial \ln\omega }=-\frac{\beta \gamma \Delta \varepsilon_{HN}{\left(\omega \tau_{HN}\right)}^{\beta }\cos\left(\frac{\beta \pi }{2}\right)\left(-\left(1+\gamma \right)\Psi \left(\omega \right)\right)}{{\left[1+2{\left(\omega \tau_{HN}\right)}^{\beta }\cos\left(\frac{\beta \pi }{2}\right)+{\left(\omega \tau_{HN}\right)}^{2\beta }\right]}^{\frac{1+\gamma }{2}}}$$

where Ψ(ω) is

(8) $$\Psi \left(\omega \right)=\arctan\left[\frac{\sin\left(\frac{\beta \pi }{2}\right)}{{\left(\omega \tau_{HN}\right)}^{-\beta }+\cos\left(\frac{\beta \pi }{2}\right)}\right]$$

**Figure 12:**
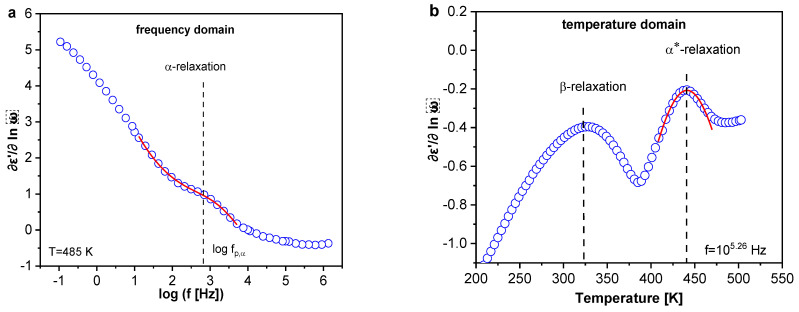
(**a**) Analysis of the α-relaxation in the frequency domain using $\frac{\partial \varepsilon^{\prime }\left(\omega \right)}{\partial \ln\omega }$ at T = 485 K, where the red line indicates the $\frac{\partial \varepsilon_{HN}^{\prime }}{\partial \ln\omega }\;$ fit; and (**b**) analysis in the temperature domain using $\frac{\partial \varepsilon^{\prime }\left(\omega \right)}{\partial \ln\omega }\;$ at f = 10^5.26^ Hz, where the red line is the Gauss fit to the curve.

The isothermal scans of the α-relaxation were analyzed by fitting $\frac{\partial \varepsilon_{HN}^{\prime }}{\partial \ln\omega }\;$to the derivative of ε′ and the relaxation rates for the α-relaxation were extracted (Figure 12a). The data can also be analyzed in the temperature domain (TD) at a fixed frequency (isochronal scans), where an additional relaxation process to the α-relaxation was observed, denoted as α*-relaxation. The peak maxima location was obtained employing a Gaussian fit to these data. However, a Lorentzian function or a parabola could also be used to analyze the isochronal data. The relaxation map for these two relaxation processes (Figure S10) illustrates that the temperature dependence of the relaxation rates of the α*-relaxation (temperature domain, isochronal representation) deviates strongly from that of the α-relaxation (frequency domain, isothermal scans). This result indicates the different molecular origins of both processes. These two processes were found at temperatures above the calorimetric glass transition region (at high frequencies). For other nanocomposite systems, an additional relaxation process related to some molecular mobility of the RAF has been previously discussed [12,78,79]. Through BDS, it was shown that RAF is not completely immobile, and a localized relaxation process can be found, faster than the bulk relaxation process with an Arrhenius-like temperature dependence. However, this cannot be the origin for either the α*-relaxation or the α-relaxation because this relaxation process was also observed for the pure epoxy. The presence of two segmental relaxations (two dynamic glass transitions) was observed by some authors [27,28] for epoxy-based systems. These processes were assigned to the structural heterogeneity of the epoxy-based matrix. Regions with lower crosslinking density would have a weaker temperature dependence compared to regions with a higher crosslinking density. Thus, α*-process was assigned to these regions. Accordingly, the α-relaxation is then linked with regions with higher crosslinking density.

To obtain a better understanding of the segmental fluctuations, the calorimetric relaxation data were added to the dielectric data. Interestingly, the TMDSC, TMFSC and α*-relaxation data have the same temperature dependence of their relaxation rates and can be described by one VFT function. This agreement indicates that the two calorimetric techniques and the isochronal scans (temperature domain) of BDS probe the same process. The process found in the frequency domain of BDS does not follow this dependency, meaning that the α-relaxation was not detected by TMDSC and TMFSC. However, as suggested in [29], the relaxation rates of the α-process can be directly compared to the thermal relaxation data estimated from the static thermal measurements. Therefore, FSC measurements at different heating rates (10^2^–10^4^ K s^−1^) were performed on the samples. Similar to conventional DSC, the step in the heat flow curves was fitted with a sigmoidal function. Next, the fit function was differentiated with respect to temperature and *T_g_* was taken as the maxima of the resulting peak. The heating rate from the static FSC data can be transformed to a thermal relaxation rate the frame of the fluctuation model of Donth [80] by

(9) $$f_{p}=C\;\frac{\dot{T}}{2\pi \Delta T_{g}}$$

**Figure 13:**
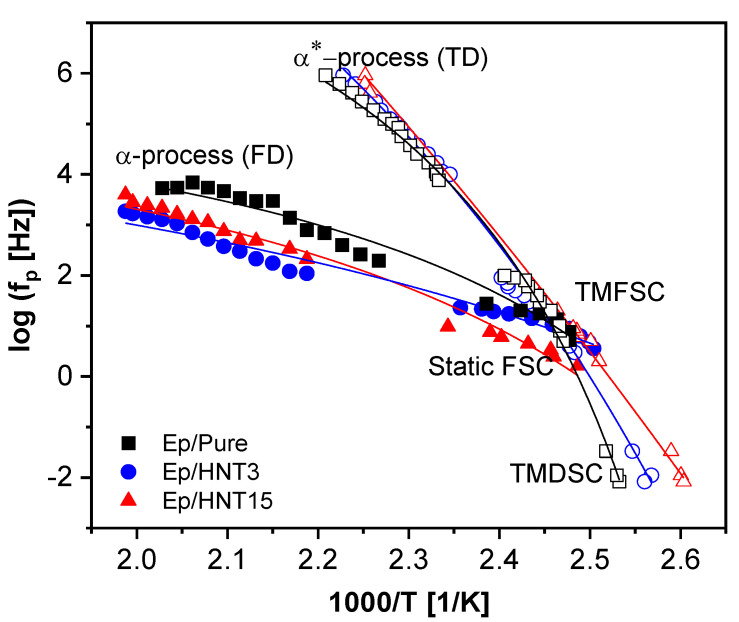
Combined map for pure epoxy and nanocomposites (Ep/HNT3 and Ep/HNT15). The lines are VFT fits to the different data sets.

*C* is a constant with a value close to one, $\dot{T}$ is the heating rate and Δ*T_g_* is the width of the glass transition. The width of the glass transition was taken as the difference between the onset and end set of the glass transition. In addition, according to the fluctuation approach, the square root of Δ*T_g_*^1/2^ should be linearly dependent on the glass transition temperature [81]; therefore, a linear regression line was fit to the data (Figure S11). From the regression line, the averaged Δ*T_g_* values were calculated and taken for each glass transition temperature and used in Equation (9) to reduce the scattering [28]. A combined relaxation map of the dielectric data and all calorimetry data is given in Figure 13, for the pure epoxy and 3 and 15 wt.% HNT. This figure shows that dielectric data obtained from the isothermal analysis agree in their temperature dependence with the relaxation rates obtained by FSC. As stated above, one VFT fit was used for the data of the α*-relaxation and the SHS data. The static FSC data do not follow the temperature dependence of the other calorimetric relaxation rates. One VFT fit was used for the thermal relaxation rates from the static FSC and the dielectric α-relaxation, indicating that they have the same molecular origin. A similar behavior was observed for an Epoxy/LDH nanocomposite system [29]. Interestingly, the static FSC data curves at lower frequencies seem to show a crossover behavior from the α-process at higher frequencies (higher temperatures) to the α*-process at lower frequencies (lower temperatures). This crossover behavior might indicate that in the static calorimetric measurements the material response for the considered system is always due to the slower process (lower relaxation rate at a given temperature).

### 3.2. Epoxy Nanocomposite with Modified Halloysite Nanotubes (Ep/m-HNT)

The HNT were modified with PDA and ultrafine iron nanodots to improve the interactions with the epoxy [36]. The hydroxyl groups of PDA might allow for a better interaction with the matrix by forming hydrogen bonds with the epoxide groups of the DGEBA. The m-HNT nanocomposites were investigated with a focus on the structure, molecular mobility and vitrification of this system in comparison to the nanocomposite with unmodified HNT in order to understand the effect of the surface modification. 

#### 3.2.1. X-ray Scattering

As for the unmodified HNT and corresponding PNCs, the structure of the modified filler and morphology of the related nanocomposites was investigated using X-ray scattering, as discussed above. As shown for m-HNT in Figure S12, in comparison to unmodified HNT, the crystal structure of the filler was not affected by the modification. Moreover, for Ep/m-HNT6 and Ep/m-HNT15, the scattering pattern in the WAXS region of the matrix (broad peaks related to carbon–carbon correlations) resembles that of the PNCs reinforced with unmodified HNT. This indicates that X-ray scattering does not detect differences in the matrix structure, compared to Ep/HNT, related to the nanofiller modification. The main difference between HNT and m-HNT is the larger outer diameter of the cylinder (D_outer_) of 113 nm for m-HNT in comparison to 107 nm for the HNT. The increase of D_outer_ for the modified particles is due to an additional layer of PDA on the external particle surface. From the difference of the two values, one can conclude that the thickness of the PDA layer is about 3 nm. Similar to Ep/HNT, for the two PNCs (Ep/m-HNT6 and Ep/m-HNT15), the D_outer_ of m-HNT increases when embedded in the matrix, compared to the pure particles (113 nm, m-HNT; ~126.7 nm, PNCs). However, for Ep/m-HNT, the increase of D_outer_ is smaller than for Ep/HNT because the PDA coating partially prevents the intercalation of small molecules into the cylinder rolls.

#### 3.2.2. TEM

**Figure 14:**
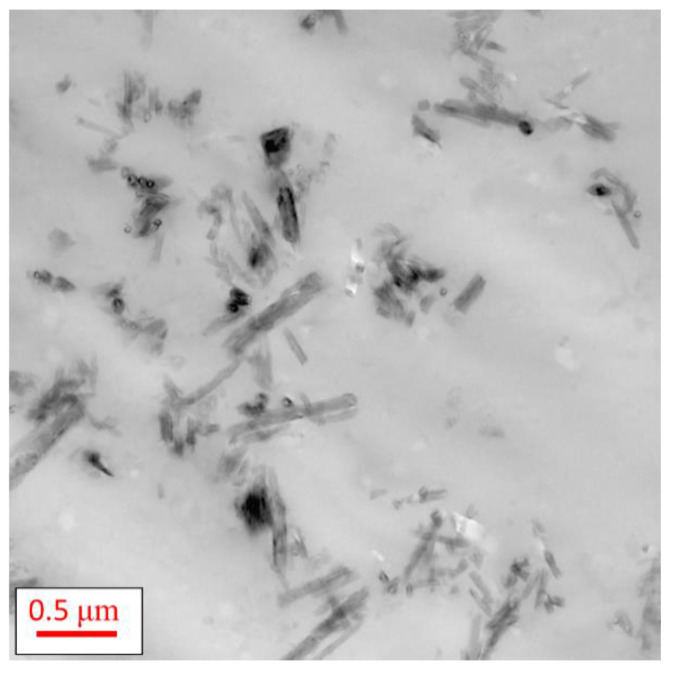
TEM image for Ep/m-HNT15 at a magnification of 0.5 µm.

A TEM image for the sample Ep/m-HNT15 is given in Figure 14 as an example. Although some clusters still exist in the nanocomposites, the m-HNT nanofillers are better dispersed compared to the nanocomposites containing the unmodified HNT (see Figure 4). This is observed for all concentrations of m-HNT.

#### 3.2.3. Calorimetry

**Figure 15:**
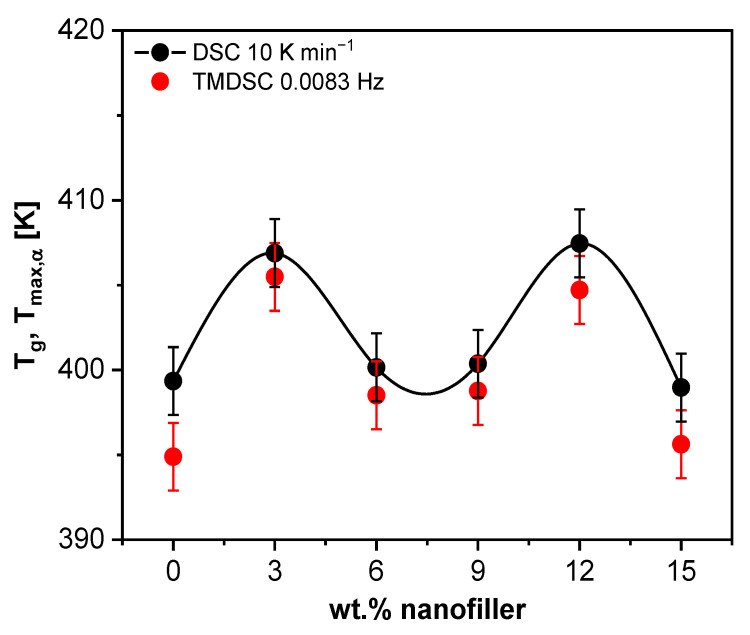
Glass transition temperature as a function of m-HNT content estimated by DSC and TMDSC. The line is a guide to the eyes.

The estimated *T_g_* values by DSC show a non-monotonous concentration dependence (see Figure 15). Nevertheless, in comparison to the Ep/HNT system, for the m-HNT containing system, the glass transition temperature values for the nanocomposites do not decrease compared to that of the pure epoxy. For three concentrations of m-HNT (6, 9 and 15 wt.%), the *T_g_* values are similar to the pure epoxy, whereas, for 3 and 12 wt.%, *T_g_* is ca. 8 K higher than that of the pure epoxy. This behavior is not easy to understand. As for the unmodified system, a competition between mobilization of the segments due to the nanofiller-related changes in crosslinking density and immobilization related to an interphase formation is expected. Different from the Ep/HNT system, a siloxane-related plasticization effect is not relevant for this system, due to the surface modification. Moreover, the immobilization effect of the segments at the filler–matrix interface is expected to be stronger because of a possible formation of hydrogen bonds between the PDA amine hydroxyl groups and the hydroxyl groups of the epoxy [82,83]. The immobilization might be counterbalanced by a mobilization effect due to a decrease in the crosslinking density related to the incorporation of nanofillers. This would result in a *T_g_* value which is similar to that of the pure epoxy. The increase of the *T_g_* for 12 wt.% of m-HNT might be discussed by a kind of percolation of the RAF phase which has a maximum for 12 wt.% (see below). For 15 wt.% m-HNT, this percolation effect is overcompensated by a further decrease in the crosslinking density. In addition, since the surface modification results in a better dispersion of the nanofillers in the epoxy matrix, there exists less amount of an HNT-rich phase, as expected for the unmodified system. Thereby, the discussion applied for the HNT embedded in a continuous matrix phase described for Ep/HNT15 would not be applicable for higher nanofiller content for the m-HNT samples. This is shown in Figure 15, where for Ep/m-HNT15 the *T_g_* is comparable to the unfilled epoxy value.

#### 3.2.4. Specific Heat Spectroscopy

The reversing specific heat capacity obtained from TMDSC was used to calculate the frequency dependent glass transition temperature, *T_max,α_*, plotted as a function of filler content in Figure 15 at f = 0.0083 Hz. The *T_max,α_* values show a similar non-monotonous concentration dependence as the DSC data. 

##### Interfacial Properties

**Figure 16:**
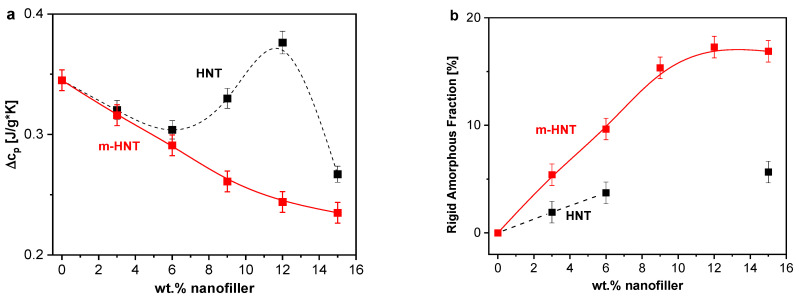
(**a**) Δc_p_ and (**b**) RAF as function of the filler content for m-HNT (red) and HNT (black). Line are guides for the eyes.

In addition, from the reversing specific heat capacity, the calorimetric strength and amount of RAF were determined as described above. Both Δc_p_ and RAF are depicted as a function of m-HNT content in Figure 16. The Δc_p_ decreases systematically with increasing nanofiller content. The immobilization of segments by the nanofillers due to interface formation is the dominant effect for all concentrations of the nanofiller resulting in a decreased calorimetric strength. This behavior is confirmed as the amount of RAF increases and reaches a maximum value at ca. 12 wt.% m-HNT. The observed maximum can be taken as an indication that agglomerates are formed in the system, as discussed for an epoxy filled with Boehmite [27]. This would reduce the amount of interfacial area available for RAF formation, compared to the dispersed nanofillers. For comparison, the data for the system containing the unmodified nanofiller is included in Figure 16. This comparison demonstrates the different behavior of both nanocomposites systems. The amount of RAF formed for Ep/m-HNT is essentially higher than that of Ep/HNT. The concentration dependence of RAF for Ep/m-HNT increases for all concentration of m-HNT, indicating a significant interphase formation. The different RAF formation for both systems is also reflected in the different concentration dependence of Δc_p_. However, the application of the three-phase model is limited because the reduction in crosslinking density is not taken into account. Therefore, only lower limits of RAF can be estimated.

##### Segmental Dynamics

The relaxation rates from TMDSC and TMFSC were also estimated as for the unmodified HNT system and plotted in the activation plot (Figure S15). A similar temperature dependence as for the unmodified system was observed for both methods. The data can be described by one VFT function for each sample. The calorimetric techniques probe the relaxation processes related to entropy fluctuations. Thereby, the relaxation plots were further extended with data from BDS to gain a complete picture of the segmental dynamics.

#### 3.2.5. Broadband Dielectric Spectroscopy

**Figure 17:**
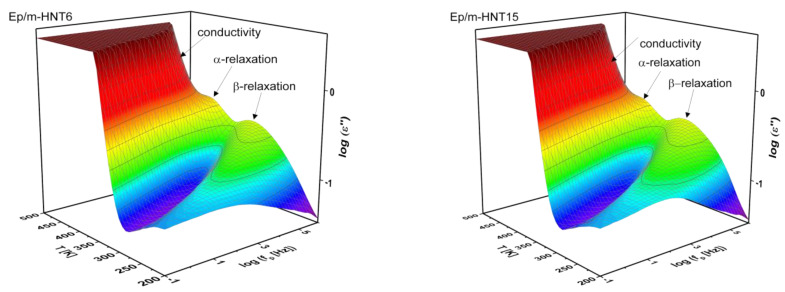
3D plots of the dielectric loss spectra as a function of frequency and temperature for Ep/m-HNT6 and Ep/m-HNT15.

BDS was employed to study the molecular mobility of the m-HNT PNCs similar to the unmodified HNT. 3D dielectric loss spectra for the samples with 6 and 15 wt.% m-HNT are depicted in Figure 17. The segmental relaxation (α-relaxation), localized fluctuations (β-relaxation) and conductivity contribution were observed to be similar to Ep/HNT. The found relaxation processes were analyzed analogously to that for the pure and Ep/HNT samples. As stated above, conductivity is a parasitic effect and is be further addressed.

**Figure 18:**
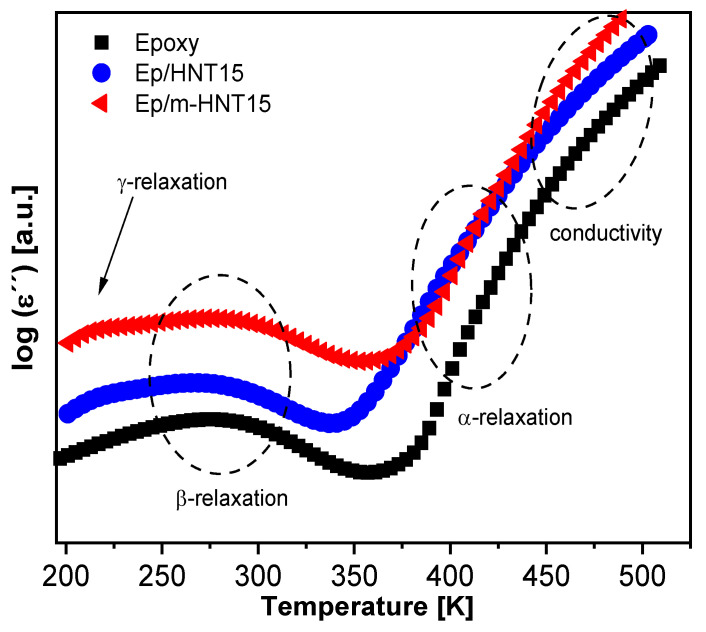
Dielectric loss spectra at a frequency of 1000 Hz for selected samples. Black squares, Ep/Pure; blue circles, Ep/HNT15; red triangles, Ep/m-HNT15.

The isochronal data for Ep/m-HNT15 was plotted alongside the pure epoxy and Ep/HNT15 sample in Figure 18 for a direct comparison of both systems. Similar to the unmodified system, the γ-processes is more pronounced for Ep/m-HNT15 than for pure epoxy, due to more dangling ends (unreacted end groups) in the matrix. Further, the β- and α-process are observed at similar positions for all samples, however a more detailed discussion is given below. The interfacial polarization effects polarizations effects seem to be more pronounced for Ep/m-HNT compared to Ep/HNT. Due to the better dispersion of the nanofiller more surface is available for the blocking of charges.

##### Localized Molecular Fluctuations

As discussed for the system with the unmodified nanofiller, the β-relaxation can be analyzed by fitting a single HN function to the data at higher temperatures, where at lower temperatures the low intensity γ-relaxation was observed, and there the data were fitted by the sum of two HN-functions.

**Figure 19:**
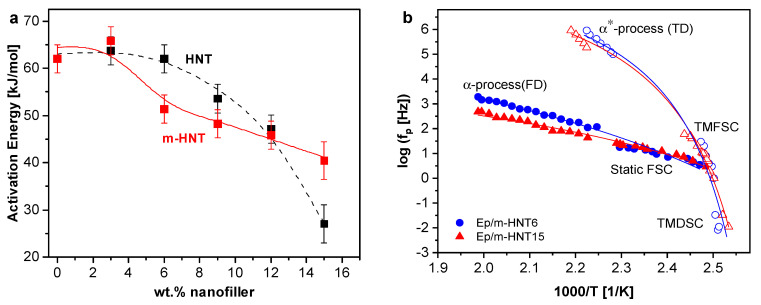
(**a**)The activation energy for β-relaxation as a function of wt.% m-HNT (red) and HNT (black). The lines are a guide to the eyes. (**b**) Combined relaxation map for 6 and 15 wt.% m-HNT. The lines are fits of the VFT equation to the corresponding data.

The relaxation rate values were extracted and plotted in the relaxation map. The activation map for these local relaxation processes is plotted in Figure S17 and the estimated activation energy for the β-relaxation is depicted in Figure 19a. The m-HNT system shows a decrease of the activation energy but with a different concentration dependence compared to the HNT system. As discussed above for the unmodified HNT samples, the decrease in E_a_ is a result of an increase in free volume caused by the decrease in crosslinking density. The incorporation of nanofiller reduces the crosslinking density, resulting in a higher free volume. The different concentration dependence found for the activation energy of the β-relaxation also reflects the difference in the interaction of the two nanofillers with the epoxy matrix.

##### Segmental Dynamics

For the segmental dynamics, the conduction-free analysis was also employed as for the system with the unmodified filler. $\frac{\partial \varepsilon_{HN}^{\prime }}{\partial \ln\omega }$ was used to analyze the α-relaxation in the frequency domain and the α*-relaxation in the isochronal domain (temperature domain). The activation map for both relaxations is shown in Figure S18, and, as for the unmodified system, the temperature dependence of the α*-relaxation deviates strongly from the α-relaxation, which indicated different molecular origins for both processes. For m-HNT, the combined α-relaxation plot with the results from the TMDSC, TMFSC, static FSC (fluctuation model, Equation (9)) and BDS is shown in Figure 19b. For clarity, only 6 and 15 wt.% m-HNT are plotted. Similar temperature dependencies are observed for the modified HNT system, compared to that of the modified samples. Again, one VFT function is used for the description of the dielectric α*-relaxation and the SHS data (TMDSC and TMFSC) and a second VFT fit for the α-relaxation and the thermal relaxation rates deduced from static FSC. As for the unmodified system, the two processes are assigned to the structural heterogeneity of the system. The regions with higher crosslinking density to the α-process and regions with lower crosslinking density to α*-process. The m-HNT system does not show a pronounced crossover at lower frequencies (lower temperatures) for the thermal relaxation rates.

## 4. Conclusions

In this study, epoxy nanocomposites, based on DGEBA cured with DETA with varying amounts of pristine and surface modified (PDA, iron nanodots) HNT nanofillers, were investigated for the first time using a combination of X-ray scattering, advanced calorimetry, specific heat and broadband dielectric spectroscopy. The morphology was first investigated by SAXS and WAXS. For the pure epoxy, three broad peaks in the WAXS region were found, related to the inherent spatial heterogeneity of the crosslinked network. A similar behavior was found for the nanocomposites. The SAXS data were analyzed using the Monte Carlo fitting method to obtain the size distributions of the nanofillers. The outer diameter of HNT and m-HNT was found to increase when embedded in the matrix, compared to the pure particles, due to the intercalation of resin and/or hardener in the cylinder rolls prior to the crosslinking reaction. The surface modification also resulted in a larger outer diameter of the filler by ca. 6 nm due to an additional layer of PDA on the external particle surface.

The glass transition behavior was elucidated using DSC measurements. For both HNT and m-HNT, the concentration dependence of the glass transition behavior was due to two competing mechanisms: (I) the mobilization of the nanofiller due to the reduction of crosslinking density; and (II) the formation of an immobilized region at the filler–matrix interphase. For the HNT samples, two additional factors were also considered: mobilization of the matrix due to the plasticizing effect related to the siloxane groups of HNT and an immobilization effect for highly loaded samples due to the formation of HNT- and epoxy-rich regions. For the Ep/HNT system, the *T_g_* for each concentration was found to be lower than for the pure epoxy, showing a decrease down to 12 wt.% due to the mobilization of the matrix being a dominant effect. For the highest concentration, an increase of *T_g_* was observed due to the additional confinement on the matrix in the epoxy-rich region. On the contrary, for m-HNT no reduction of the glass transition temperature was observed, with higher *T_g_* values for some concentrations, compared to that of the pure epoxy. This indicates that firstly no plasticization is present and secondly a stronger interaction between the matrix and the nanotubes is expected due to a hydrogen bonding between the hydroxyl groups of PDA with DGEBA. In addition, due to the stronger NP–matrix interaction, the m-HNTs are less agglomerated than HNTs, and, thus, immobilization due to the nanoparticle-rich region is not observed.

TMDSC was used to investigate the molecular mobility and structure, including the immobilized fraction at the matrix/particle interface (RAF) of the samples. For the unmodified HNT, the concentration dependence of the specific heat capacity showed that the discussed competition of mobilization and immobilization of the NPs led to both a decrease and an increase in the calculated values of calorimetric strength (Δc_p_) compared to that of the pure epoxy. Above the concentration of 6 wt.% HNT, the three-phase model cannot be used to describe the interphase, as the mobilization of the matrix is the dominating effect. For Ep/m-HNT, the calorimetric strength decreases systematically with increasing filler content, indicating that the interphase formation was dominant over matrix mobilization for all concentrations. RAF showed a systematic increase with increasing NPs concentration and reached values up to ~16%. The higher RAF amounts for Ep/m-HNT compared to Ep/HNT are a result of the surface modification related to stronger matrix–NP interaction and more homogenous distribution of nanofillers in the epoxy matrix.

Further, the molecular mobility was studied using broadband dielectric spectroscopy. For both nanocomposites at temperatures below the *T_g_*, β-relaxation and γ-relaxation were found and attributed to the rotational motions of the phenyl rings of DGEBA and the local motions of unreacted components, respectively. The activation energy was estimated for the β-relaxation, showing a decrease with increasing nanofiller content for both systems. The decrease in E_a_ is a result of an increase in free volume caused by the decrease in crosslinking density. At temperatures above the glass transition region, α-relaxation was found in the frequency domain (isothermal scans). An additional process was found in the temperature domain (isochronal scans) at high frequencies, named α*-process, which is also related to segmental dynamics. The presence of the two processes indicated a structural heterogeneity of epoxy-based materials, as implied by X-ray scattering. The two relaxations were assigned to regions in the matrix with different crosslinking density. The α*-process was assigned to regions with lower crosslinking density and the α-process to higher crosslinking density. The complete activation map was constructed by also including the calorimetric relaxation rates (TMDSC and TMFSC) and thermal relaxation rates calculated from static FSC measurements. Two different VFT functions were fitted, one for the TMDSC, TMFSC and dielectric α*-process (frequency domain) and the other for the static FSC and the dielectric α-process (temperature domain). Interestingly, thermal relaxations from static FSC show a crossover behavior for Ep/HNT from α-process at higher frequencies to α*-process at lower frequencies, not observed for the modified system.

## Data Availability

The data presented in this study are available on request from the corresponding author.

## Supplementary Materials

The following are available online at https://www.mdpi.com/article/10.3390/polym13101634/s1, Figure S1: Mass loss as a function of temperature.; Figure S2: X-ray scattering patterns of the pure epoxy, halloysite nanotubes and nanocomposites.; Figure S3: Volume-weighted size distribution from Monte Carlo fitting methods for the Ep/HNT6 and Ep/HNT15 samples, as well as the HNT nanofiller.; Figure S4: Simulations for D_sheet_ (sheet thickness) (0.25, 0.5 and 1 nm), D_lumen_ (lumen size) (3, 5 and 7 nm), D_length_ (tube length) (50, 100 and 150 nm), and D_outer_ (outer diameter) (11.5, 13 and 16 nm) to show the fit changes with variations in length parameters. Figure S5: TEM images for Ep/HNT6 at lower magnification and higher magnification.; Figure S6: Heat flow curve for Ep/HNT3 with a heating rate of 10 K min^−1^.; Figure S7: Flash DSC data for EP/HNT6 with various heating rates.; Figure S8: Modulus of the specific heat capacity $|c_{p}^{*}|\;$for the Ep/HNT3 using an isotherm time of 120 s.; Figure S9: Normalized modulus of the heat capacity $\left|C_{p}^{*}\right|$ at 20 Hz for Ep/HNT nanocomposites and the pure epoxy.; Figure S10: Combined α-relaxation plot with the α- and α*-processes.; Figure S11: The square root of the glass transition width as a function of T_g_.; Figure S12: X-ray scattering patterns of the pure epoxy, modified halloysite nanotubes and nanocomposites (Ep/m-HNT6 and Ep/m-HNT15).; Figure S13: Volume-weighted size distribution from Monte Carlo fitting methods for the Ep/m-HNT6 and Ep/m-HNT15 samples, as well as the m-HNT nanofiller.; Figure S14: Heat flow curves for the pure epoxy and the modified HNT samples at 10 K min^−1^.; Figure S15: Combined calorimetric activation plot for the TMFSC and TMDSC.; Figure S16: Dielectric loss spectra at a frequency of 1000 Hz for selected samples.; Figure S17: Activation plot for the β-relaxation and γ-relaxation.; Figure S18: Combined α-relaxation plot with the α- and α*-process.; Table S1: McSAS fit average values for the D_sheet_, D_lumen_ and D_outer_ for the nanofillers and the epoxy nanocomposites.
