# Supplementary material for: Calorimetric and Dielectric Investigations of Epoxy-Based Nanocomposites with Halloysite Nanotubes as Nanofillers
Source: Polymers (Basel). 2021 May 18;13(10):1634. doi: 10.3390/polym13101634 (PMC8157573; doi:10.3390/polym13101634)
Supplement: Supplementary file 1 [file polymers-13-01634-s001.zip › polymers-1211773-supplementary.pdf]

Supplementary Materials

Calorimetric and dielectric investigations of epoxy nanocomposites with unmodified and modified halloysite nanotubes as nanofilllers

Hassan Omar [a], Glen J. Smales [a], Sven Henning [b], Zhi Li [c,#], De-Yi Wang [c], Andreas Schönhals [a], Paulina Szymoniak [a,*]

[a] Bundesanstalt für Materialforschung und - Prüfung (BAM), Unter den Eichen 87, 12205 Berlin Germany

[b] Fraunhofer-Institut für Mikrostruktur von Werkstoffen und Systemen (IMWS), Walter-Hülse-Str. 1, 06120 Halle, Germany

[c] IMDEA Materials Institute, C/Eric Kandel 2, 28906 Getafe, Madrid, Spain

[#] Current address: China-Spain Collaborative Research Center for Advanced Materials, School of Materials Science and Engineering, Chongqing Jiaotong University, Chongqing, 400074, China

Epoxy/HNT nanocomposites

Thermogravimetric Analysis (TGA) – Mass loss curves

TGA was performed using a STA7000 Series Thermogravimetric Analyzer (Hitachi, Chidoya, Japan), employed with a horizontal dual balance beam, enabling a microgram-level weight change detection. Samples of 5.98 – 6.37 mg were heated in alumina pans from room temperature up to 1250 K at a heating rate of 10 K min^{-1}. Nitrogen was used as a purge gas. The inoriganic residue (mass of the HNT nanofiller) was calculated by subtracting the mass of the pure epoxy residue from the overall residue.

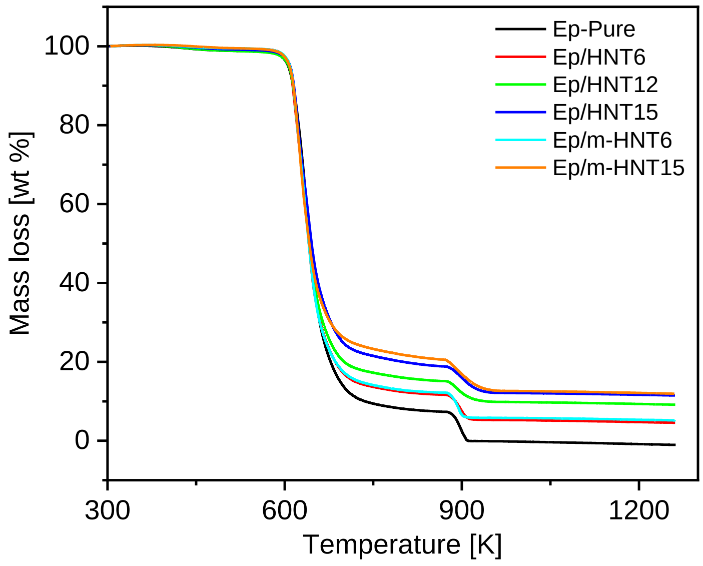

Figure S1: Mass loss as a function of temperature. The samples are heated in nitrogen from 300 to 1250 K.

X-ray scattering – raw data

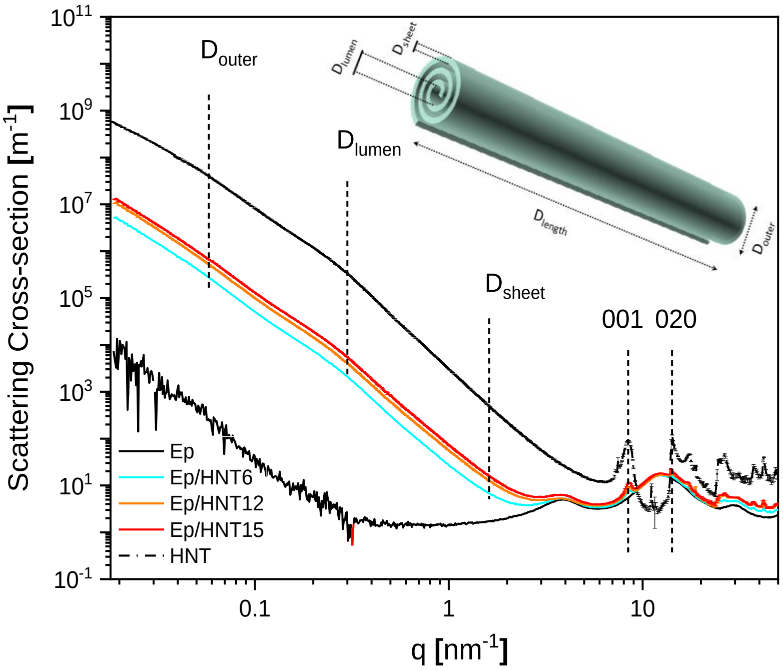

Figure S2: X-ray scattering patterns of the pure epoxy, halloysite nanotubes and nanocomposites as indicated. The inset gives a scheme of the halloysite rolled cylinder with different dimensions indicated.

X-ray scattering – McSAS fit & size distributions

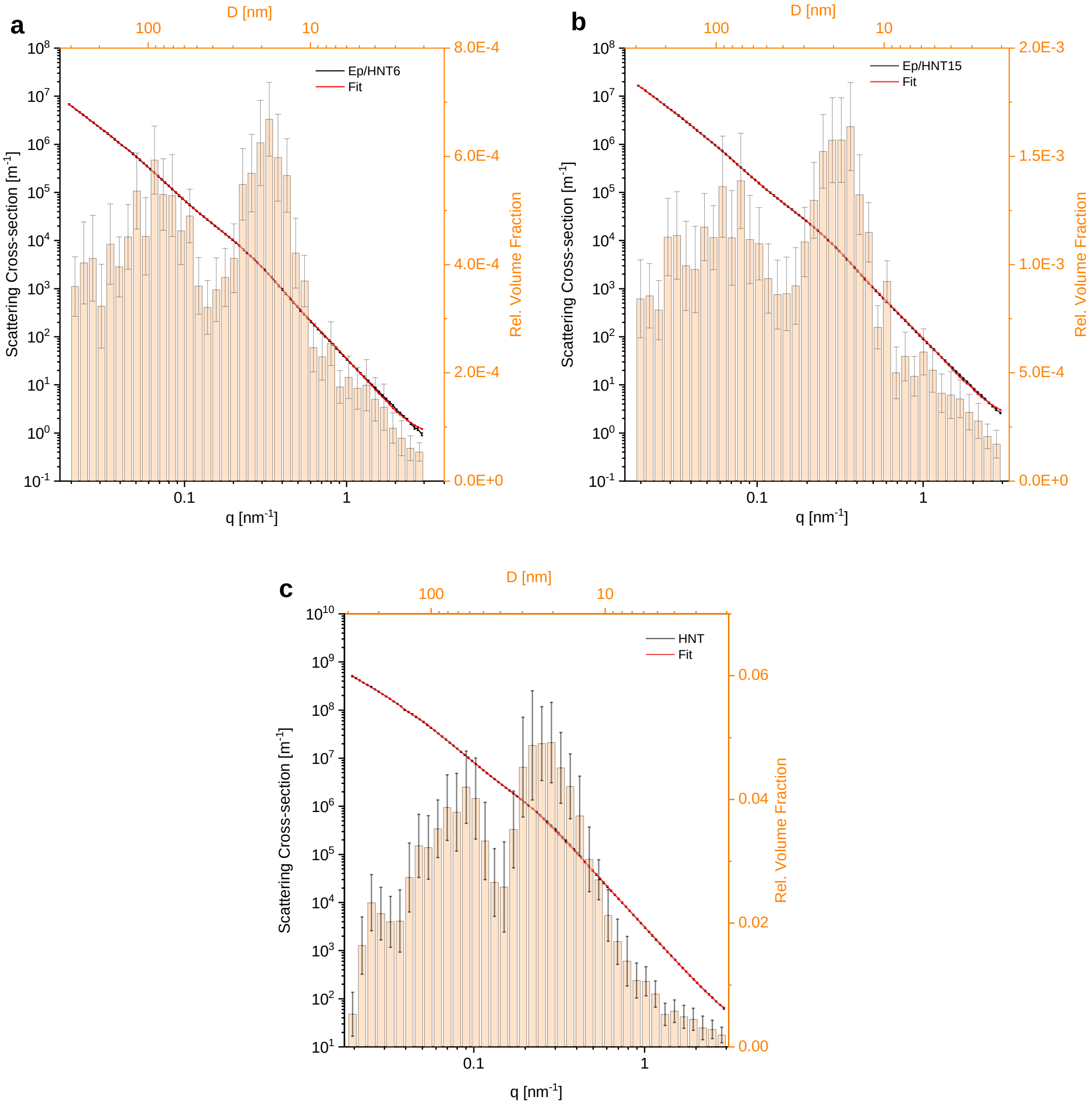

Figure S3: Volume-weighted size distribution (orange bar graphs) from Monte Carlo fitting methods for the (a) Ep/HNT6 and (b) Ep/HNT15 samples, as well as the (c) HNT nanofiller. The top X-axis is inverted to roughly show the size distribution in correlation to the features observed in the SAXS data ($D = 2\pi/q$). The fit line to the X-ray data is indicated in red and error bars are included.

X-ray scattering – simulations with different parameter changes

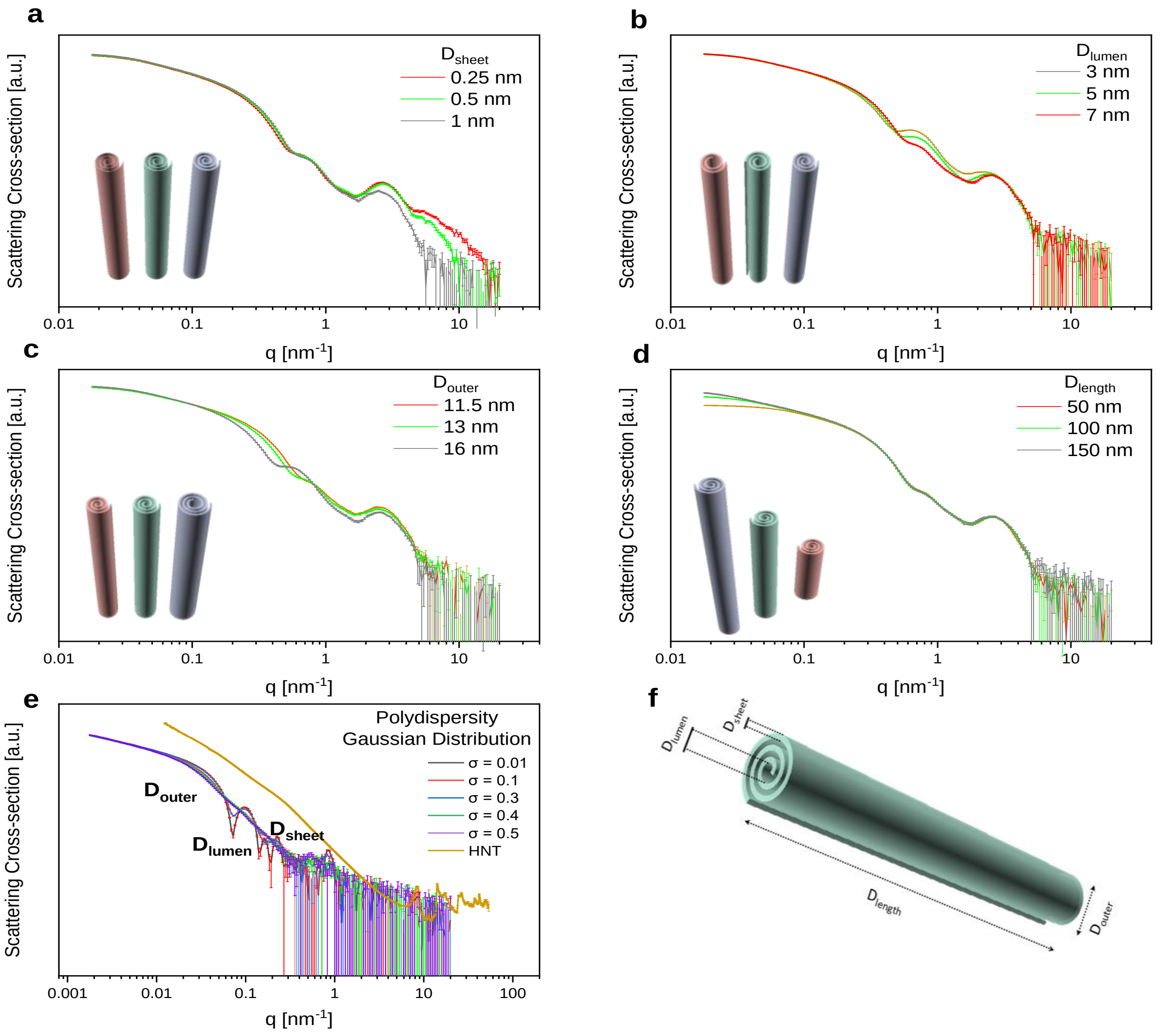

Figure S4: Simulations for (a) D_{sheet} (sheet thickness) (0.25, 0.5 and 1 nm), (b) D_{lumen} (lumen size) (3, 5 and 7 nm), (c) D_{length} (tube length) (50, 100 and 150 nm), and (d) D_{outer} (outer diameter) (11.5, 13 and 16 nm) to show the fit changes with variations in length parameters. Polydispersity (e) was also simulated as a Gaussian distribution between σ = 0.1 – 0.5. When simulating changes in D_{outer}, it is importatnt to note that both the D_{outer} and the D_{lumen} is affected (D_{lumen} = 2, 3 and 6 nm for D_{outer} = 11.5, 13 and 16 nm, respectively). Due to this changes in two features of the simulated data are observed between 0.2 – 1 nm^{-1}.

Transmission elctron microscopy – Ep/HNT6 images

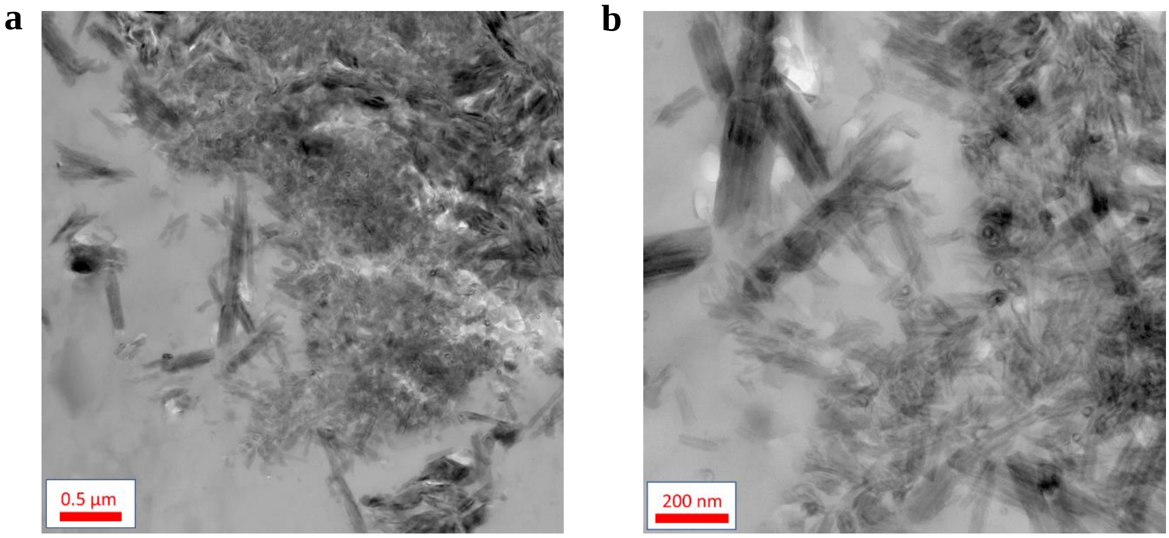

Figure S5: TEM images for Ep/HNT6 (a) lower magnification and (b) higher magnification.

Conventional DSC – analysis of the heat flow curves

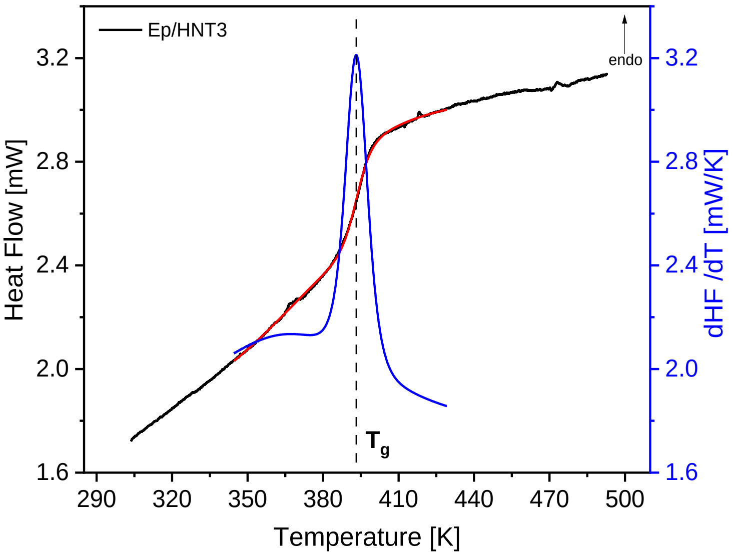

Figure S6: Heat flow curve for Ep/HNT3 with a heating rate of 10 K min^{-1}. The red curve is the sigmoidal fit to the raw data. The blue curve is the derivative of the sigmoidal fit with respect to temperature. The peak maxima of the derivative is taken as the glass transition temperature (T_g).

Flash DSC – heat flow curves at different heating rates

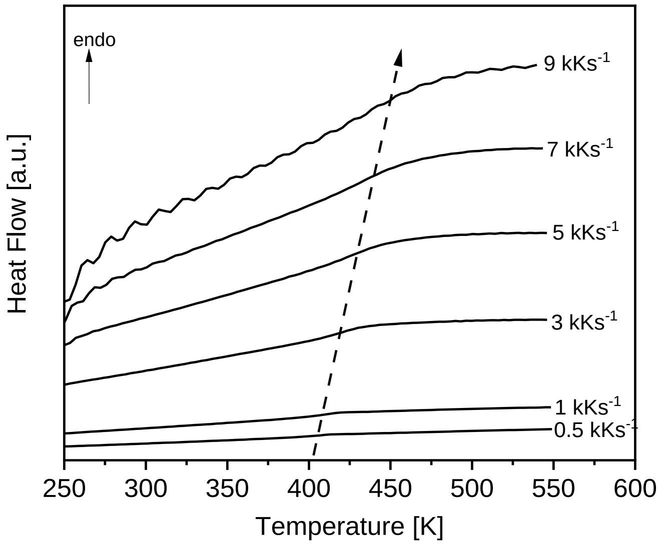

Figure S7: Flash DSC data for EP/HNT6 with various heating rates labelled. The dashed line indicates the shift of the glass transition region with increasing heating rates. The data is shifted along the y-axis for clarity.

Temperature modulated DSC – calculation of calorimetric strength

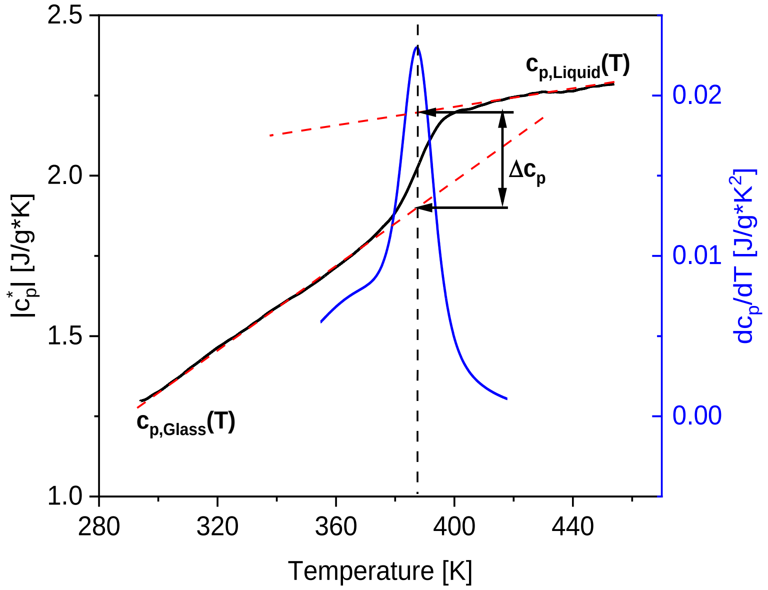

Figure S8: Modulus of the specific heat capacity $|c_p^*|$ for the Ep/HNT3 using an isotherm time of 120 s, where the calorimetric strength is the difference between the $c_{p,Liquid}$ and $c_{p,Glass}$ at T_g. The red dashed lines indicate the slope of the glassy and liquid pleteaus.

Temperature modulated FSC

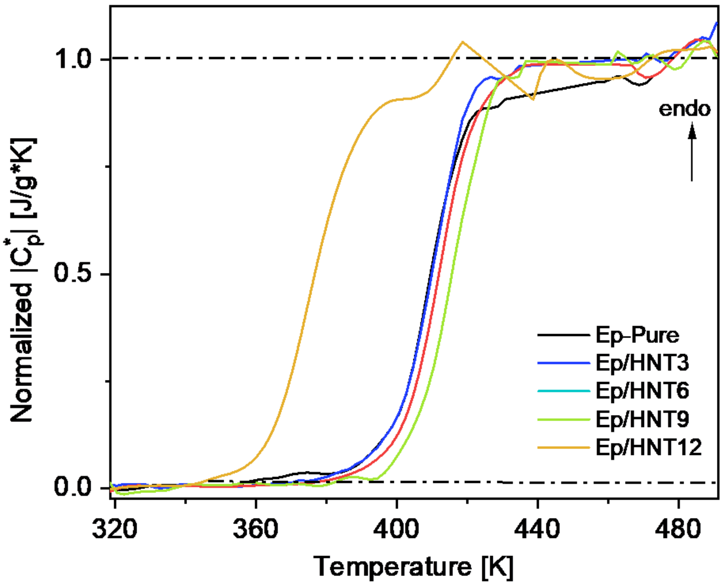

Figure S9: Normalized modulus of the heat capacity $|C_p^*|$ at 20 Hz for Ep/HNT nanocomposites and the pure epoxy

Broadband dielectric spectroscopy – combined α-relaxation activation map

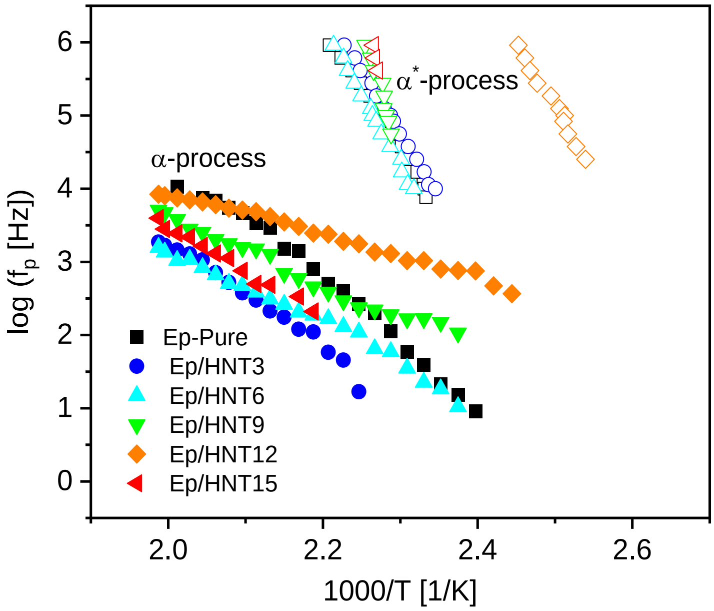

Figure S10: Combined α-relaxation plot with the α- and α*-processes labelled. Black squares – pure epoxy (Ep-Pure), blue circles – Ep/HNT3, teal triangles – Ep/HNT6, green triangles – Ep/HNT9, orange diamonds – Ep/HNT12, and red triangles – Ep/HNT15.

Flash DSC – width of the glass transition vs. T_g

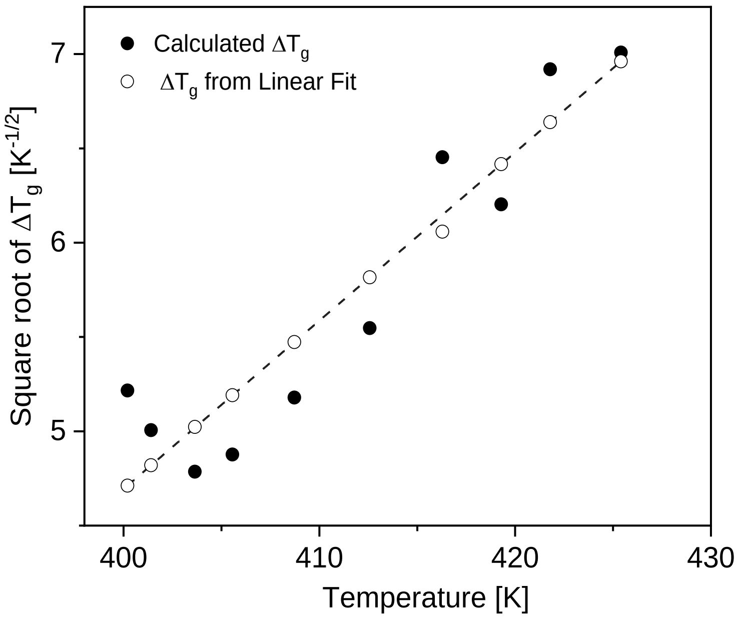

Figure S11: The square root of the glass transition width as a function of T_g. Black filled circles are the widths and dashed line is a regression line to the data. The empty circles are the data used for calculation of the thermal relaxation rates.

X-ray scattering – SAXS/WAXS for m-HNT samples

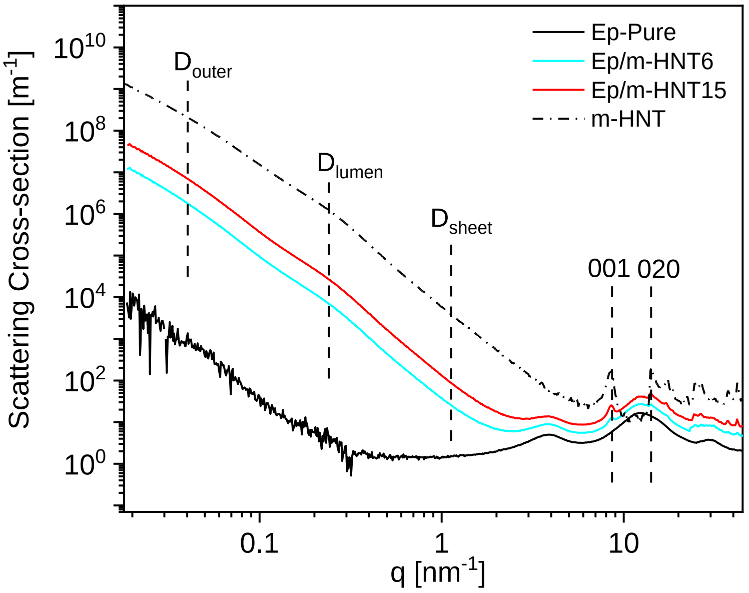

Figure S12: X-ray scattering patterns of the pure epoxy, modified halloysite nanotubes and nanocomposites (Ep/m-HNT6 and Ep/m-HNT15). The curves are shifted alongthe Y-axis for the sake of clarity and the broad peaks are labelled by their respective dimension.

Epoxy/m-HNT nanocomposites

X-ray scattering – McSAS fit and size distributions

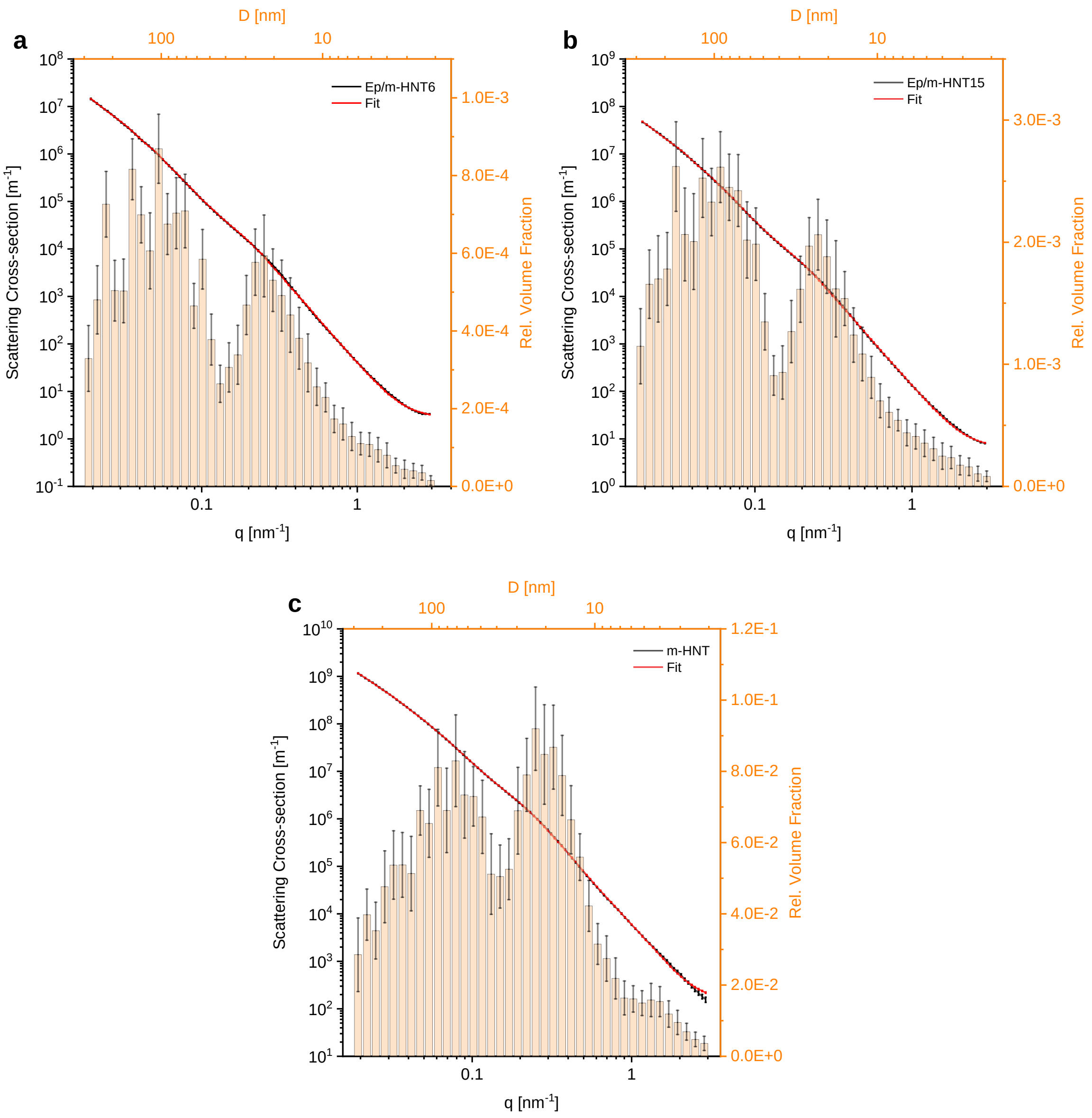

Figure S13: Volume-weighted size distribution (orange bar graphs) from Monte Carlo fitting methods for the (a) Ep/m-HNT6 and (b) Ep/m-HNT15 samples, as well as the (c) m--HNT nanofiller. The top X-axis is inverted to roughly show the size distribution in correlation to the features observed in the SAXS data (D = 2π/q). The fit line to the X-ray data is indicated in red and error bars are included

Conventional DSC – heat flow curves

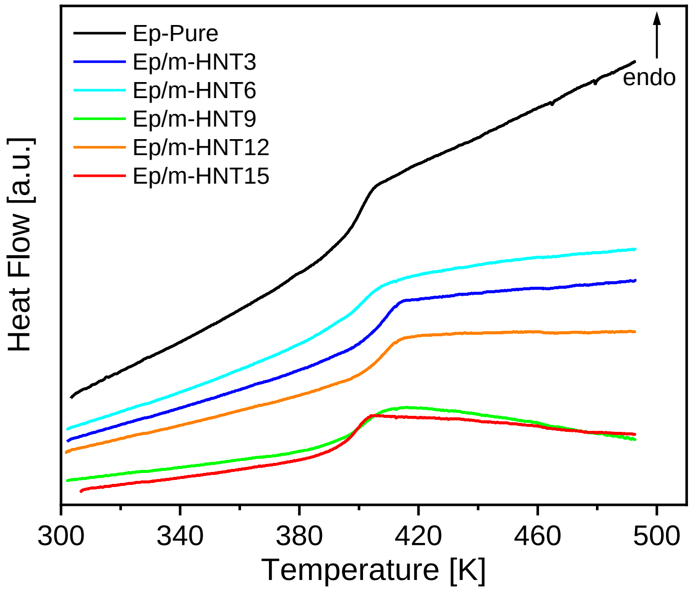

Figure S14: Heat flow curves for the pure epoxy and the modified HNT samples at 10 K min^{-1}.

Combined calorimetric activation plot

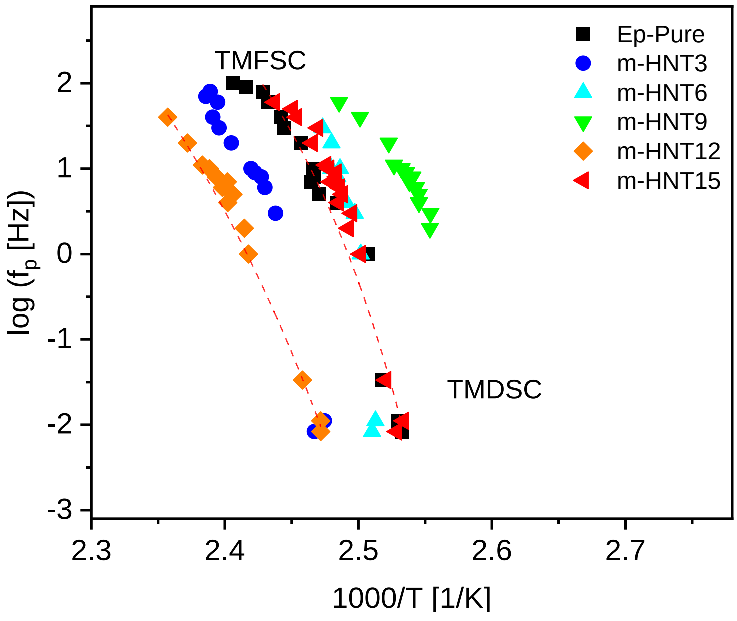

Figure S15: Combined calorimetric activation plot for the TMFSC and TMDSC. Black squares – pure epoxy, blue circles – Ep/m-HNT3, teal triangles – Ep/m-HNT6, green triangles – Ep/m-HNT9, orange diamonds – Ep/m-HNT12, and red triangles – Ep/m-HNT15. The red dashed lines are VFT fits to the TMFSC and TMDSC data to indicate the same temperature dependence obtained for these processes.

Broadband dielectric spectroscopy – dielectric loss spectra at 1000 Hz for mHNT

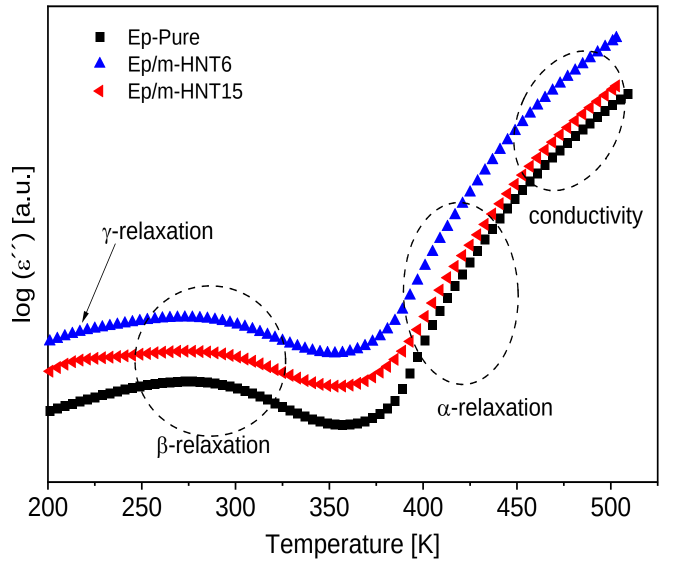

Figure S16: Dielectric loss spectra at a frequency of 1000 Hz for selected samples. Black squares – pure epoxy, teal triangles – Ep/m-HNT6, and red triangles – Ep/m-HNT15. The dielectric loss is shifted along the y-axis for sake of clarity.

Broadband dielectric spectroscopy – activation plot for β- and γ-relaxations

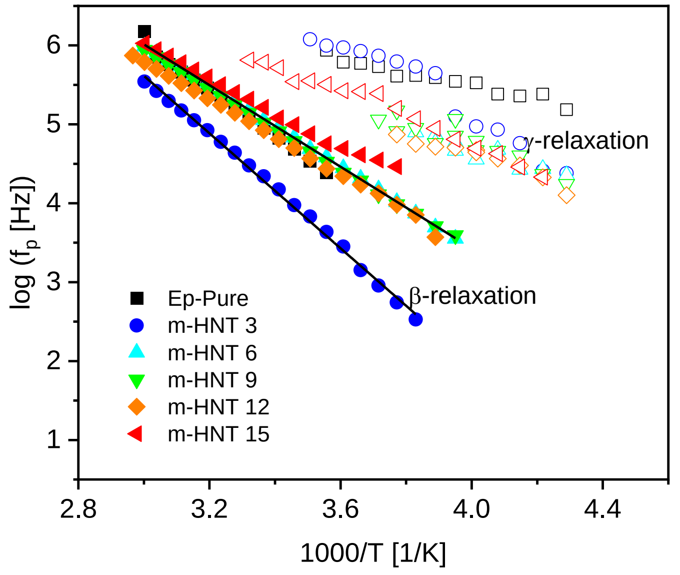

Figure S17: Activation plot for the β-relaxation (solid symbols) and γ-relaxation (open symbols). Black squares – pure epoxy, blue circles – Ep/m-HNT3, teal triangles – Ep/m-HNT6, green triangles – Ep/m-HNT9, orange diamonds – Ep/m-HNT12, and red triangles – Ep/m-HNT15. The black lines are Arrhenius fits to the β-relaxation.

Broadband dielectric spectroscopy – combined α-relaxation activation map

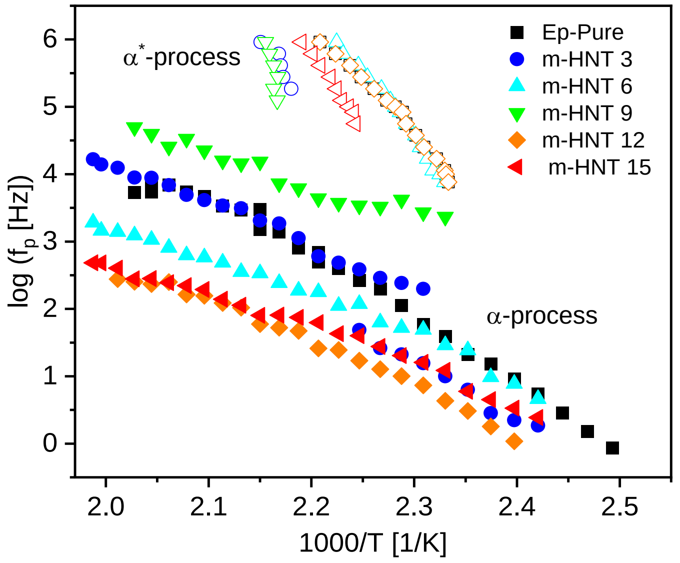

Figure S18: Combined α-relaxation plot with the α- and α*-process labelled. Black squares – Ep Pure, blue circles – Ep/m-HNT3, teal triangles – Ep/m-HNT6, green triangles – Ep/m-HNT9, orange diamonds – Ep/m-HNT12, and red triangles – Ep/m-HNT15.

Table 1 – X-ray scattering

Table S1: McSAS fit average values for the D_{sheet}, D_{lumen} and D_{outer} for the nanofillers and the epoxy nanocomposites.

	**1 - 6 nm (D_{sheet})**		**6 - 40 nm (D_{lumen})**		**40 - 300 nm (D_{outer})**	
	**Mean**	**Error**	**Mean**	**Error**	**Mean**	**Error**
**HNT**	3.836	0.05262	19.718	0.062	107	0.9244
**Ep/HNT6**	4.060	0.08226	19.252	0.145	124.04	1.0066
**Ep/HNT12**	3.946	0.04858	19.350	0.082	127.32	0.9134
**Ep/HNT15**	4.098	0.07032	19.090	0.137	124.14	1.2062
**Ep/HNTx (average)**	4.035	0.06705	19.231	0.121	125.17	1.0421
	**Mean**	**Error**	**Mean**	**Error**	**Mean**	**Error**
**m-HNT**	3.786	0.05546	19.958	1.875	112.70	1.1080
**Ep/m-HNT6**	4.222	0.13100	20.180	0.324	127.36	1.2906
**Ep/m-HNT15**	4.104	0.11942	20.360	0.386	126.34	0.7786
**Ep/m-HNTx (average)**	4.163	0.12970	20.270	0.355	126.85	1.0340
